# A genome-scale metabolic model for the denitrifying bacterium *Thauera sp*. MZ1T accurately predicts degradation of pollutants and production of polymers

**DOI:** 10.1371/journal.pcbi.1012736

**Published:** 2025-01-07

**Authors:** Diego Tec-Campos, Juan D. Tibocha-Bonilla, Celina Jiang, Anurag Passi, Deepan Thiruppathy, Cristal Zuñiga, Camila Posadas, Alejandro Zepeda, Karsten Zengler

**Affiliations:** 1 Facultad de Ingeniería Química, Universidad Autónoma de Yucatán, Mérida, Yucatán, México; 2 Department of Pediatrics, University of California, San Diego, 9500 Gilman Drive, La Jolla, California, United States of America; 3 Bioinformatics and Systems Biology Graduate Program, University of California, San Diego, La Jolla, California, United States of America; 4 Department of Biology, San Diego State University 5500 Campanile Drive, San Diego, California, United States of America; 5 Department of Bioengineering, University of California, San Diego, 9500 Gilman Drive, La Jolla, California, United States of America; 6 Center for Microbiome Innovation, University of California, San Diego, 9500 Gilman Drive, La Jolla, California, United States of America; 7 Program in Materials Science and Engineering, University of California, San Diego, 9500 Gilman Drive, La Jolla, California, United States of America; Institute for Stem Cell Science and Regenerative Medicine, INDIA

## Abstract

The denitrifying bacterium *Thauera sp*. MZ1T, a common member of microbial communities in wastewater treatment facilities, can produce different compounds from a range of carbon (C) and nitrogen (N) sources under aerobic and anaerobic conditions. In these different conditions, *Thauera* modifies its metabolism to produce different compounds that influence the microbial community. In particular, *Thauera sp*. MZ1T produces different exopolysaccharides with floc-forming properties, impacting the physical disposition of wastewater consortia and the efficiency of nutrient assimilation by the microbial community. Under N-limiting conditions, *Thauera sp*. MZ1T decreases its growth rate and accelerates the accumulation of polyhydroxyalkanoate-related (PHA) compounds including polyhydroxybutyrate (PHB), which plays a fundamental role as C and energy storage in this β-proteobacterium. However, the metabolic mechanisms employed by *Thauera sp*. MZ1T to assimilate and catabolize many of the different C and N sources under aerobic and anaerobic conditions remain unknown. Systems biology approaches such as genome-scale metabolic modeling have been successfully used to unveil complex metabolic mechanisms for various microorganisms. Here, we developed a comprehensive metabolic model (M-model) for *Thauera sp*. MZ1T (*i*Thauera861), consisting of 1,744 metabolites, 2,384 reactions, and 861 genes. We validated the model experimentally using over 70 different C and N sources under both aerobic and anaerobic conditions. *i*Thauera861 achieved a prediction accuracy of 95% for growth on various C and N sources and close to 85% for assimilation of aromatic compounds under denitrifying conditions. The M-model was subsequently deployed to determine the effects of substrates, oxygen presence, and the C:N ratio on the production of PHB and exopolysaccharides (EPS), showing the highest polymer yields are achieved with nucleotides and amino acids under aerobic conditions. This comprehensive M-model will help reveal the metabolic processes by which this ubiquitous species influences communities in wastewater treatment systems and natural environments.

## Author summary

*Thauera* sp. MZ1T actively participates in the nitrogen cycle by transforming nitrite or nitrate into molecular nitrogen. This denitrifying bacterium has been studied to unveil the degradation of aromatic compounds in different oxygen concentrations, or under denitrification, and dissimilatory reduction to ammonium, as well as to elucidate the production of complex polymers like polyhydroxyalkanoates and exopolysaccharides. Constraint-based metabolic models are systems biology tools capable of accurately predicting phenotypes of bacterial, archaeal, and eukaryotic organisms under multiple growth conditions. The metabolic model of *Thauera* sp. MZ1T, *i*Thauera861, accurately predicts growth rates of over 70 carbon and nitrogen sources (e.g. amides, alcohol derivatives, amines, amino acids, aromatic compounds, organic acids, nucleotides, etc.) under different oxygen conditions. Furthermore, *i*Thauera861 precisely simulates the effect of different C sources and C:N ratios under aerobic and anaerobic conditions on the production of polyhydroxybutyrate and six specific exopolysaccharides in *Thauera* sp. MZ1T. The model represents the first comprehensive M-model for *Thauera* sp. MZ1T to date, thus aiding the elucidation of metabolic roles in different environments of this denitrifying bacterium.

## 1. Introduction

*Thauera* sp. MZ1T is a floc-forming Gram-negative bacterium frequently found in wet soil, polluted freshwater, and in wastewater treatment facilities [1]. This facultative anaerobic bacterium belongs to the family *Rhodocyclaceae* within the β-proteobacteria and performs versatile metabolic processes that influence the environments it grows in [2]. Other members of *Rhodocyclaceae* are similarly abundant in soil, sediments, and aquatic systems [3]. The *Thauera* genus plays a crucial role in the nitrogen (N) cycle, converting inorganic N sources (ammonium, nitrate, and nitrite) into molecular N (N_2_) through denitrification [4,5]. The *Thauera* genus contains over 80 fully sequenced members (Refseq and Genbank) [6–9]. Most members of this genus can perform denitrification [4–6] and degrade aromatic compounds in the presence or absence of oxygen [10,11]. *Thauera* members such as *T*. *selenatis*, *T*. *aromatica*, and *Thauera sp*. MZ1T, can produce various polymers, e.g. polyhydroxyalkanoates (PHA), which play a key role as a carbon (C) storage molecule [9,12]. These bacteria can also make exopolysaccharides (EPS) that are involved in floc formation in wastewater treatment systems [13–16]. *Thauera* sp. MZ1T in particular contains multiple specialized oxygen-sensitive enzymes for the production of N_2_ and denitrification intermediates such as nitrous and nitric oxide [1,12,15,17]. Additionally, it can perform ammonification via dissimilatory nitrate reduction to ammonium (DNRA) using nitrite reductase under anoxic and limited N conditions, switching from oxygen to nitrate as a terminal electron acceptor [18,19]. At elevated oxygen levels, denitrification and DNRA pathways are partially or totally repressed and the organism switches back to aerobic respiration with oxygen as the terminal electron acceptor. In *Thauera* sp. MZ1T’s metabolism, oxygen and N compounds (like nitrate and nitrite) serve as terminal electron acceptors under varying environmental conditions, facilitating energy generation and enabling metabolic flexibility. This adaptability allows the bacterium to thrive in fluctuating oxygen environments common in wastewater systems. Low N conditions also trigger the biosynthesis of PHA-related compounds including polyhydroxybutyrate (PHB) as an important C storage molecule. Under high C:N ratio conditions (>5:1), *Thauera* sp. MZ1T produces PHB to sequester C within intracellular granules [12,15]. This behavior is sharply elevated with acetate as the primary C source, permitting selective PHA-related compound generation from *Thauera* sp. MZ1T [1,12,15].

*Thauera* sp. MZ1T is metabolically highly versatile and capable of growing heterotrophically with multiple C sources under aerobic and anaerobic conditions. The bacterium can assimilate various carbohydrates (e.g. glucose, fructose, galactose, sucrose), organic acids (e.g. acetate, lactate, citrate, and formic acid), alcohols (methanol, ethanol, and butanol), and aromatic compounds (e.g. toluene, xylene, various phenolic compounds, and benzoate) [1,12,15–18,20]. Under hypoxic or anoxic conditions, it can simultaneously denitrify and remove these aromatic compounds through assimilation and degradation [1,12,15–17]. Furthermore, it can assimilate important organic N compounds like urea, various amino acids (e.g. alanine, aspartate, glutamine) and nucleotide-related compounds [15,16]. In wastewater systems *Thauera* sp MZ1T produces abundant amounts of floc-forming EPS via four main intermediates: dTDP-D-N-acetylfucosamine, dTDP-L-rhamnose, UDP-D-galactose, and UDP-N-acetylglucosamine. These floc-forming EPS can significantly impact the structure and total biomass of the wastewater community [13,14,21]. *Thauera* sp MZ1T also has the versatility to produce EPS from inositol, but only under particular scenarios [12,16,17]. Its capability to assimilate various C and N compounds and promote floc formation contributes to *Thauera* sp. MZ1T’s abundance and importance in wastewater microbial communities [21,22].

Bioinformatics tools have been previously employed to elucidate genomic features and identify functional genes involved in denitrification, DNRA, PHA and EPS biosynthesis, and aromatic compound degradation to infer the metabolic potential of this versatile *Thauera* species [1,12,15,16]. However, the mechanisms by which this strain regulates these capabilities in response to specific resource conditions, and thereby influences the surrounding microbial community and its environment are still poorly characterized. Genome-scale metabolic models (GEMs) represent a fundamental approach to explore microbial metabolic functions across various conditions, allowing accurate predictions of metabolic trade-offs in response to specific environmental and resource constraints [23–27]. By simulating condition-specific metabolic pathways, GEMs support the investigation of complex microbial processes such as denitrification, polysaccharide production, and degradation of environmental pollutants [23–27]. To address this, we reconstructed a GEM for *Thauera* sp. MZ1T (*i*Thauera861) using semi-automated methods. *i*Thauera861 contains 1,744 metabolites, 2,384 reactions, and 861 genes. The initial draft model was manually refined to improve the quality of the phenotypic predictions. Using experimental data, the model was constrained under heterotrophic conditions. Over 70 C and N sources were evaluated under aerobic and anaerobic conditions to assess the accuracy of the model. Additionally, *i*Thauera861 was evaluated under oxygen- and N-limiting conditions to quantify the changes in production of PHB and EPS using multiple C substrates. The model is the first refined and validated M-model for any *Thauera* member and will aid in unraveling the impact these bacteria have on the surrounding environments. iThauera861 offers a valuable platform for studying *Thauera* sp. MZ1T metabolic pathways, with applications in optimizing PHB and EPS production critical for microbial flocculation and C storage in wastewater treatment. It enables detailed exploration of C and N utilization strategies, enhancing our understanding of the organism’s ability to degrade diverse organic compounds under varying conditions. By simulating diverse environmental scenarios, *i*Thauera861 provides insights for improving bioreactor efficiency and advancing theoretical studies on microbial community dynamics. This refined GEM serves as a relevant tool for both applied and fundamental research into the roles of *Thauera* sp. MZ1T in wastewater ecosystems.

## 2. Results

### Semi-automated metabolic network reconstruction of *Thauera sp*. MZ1T

**Fig 1:**
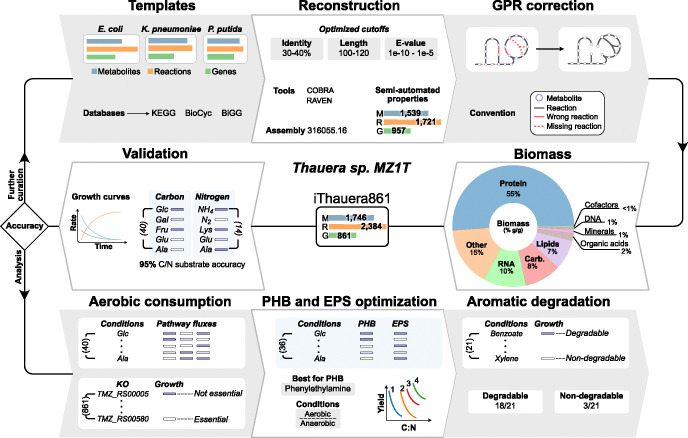
Workflow to build the metabolic model of *Thauera sp*. MZ1T using a semiautomatic approach. An initial draft M-model was reconstructed using three sets of BLASTp parameters (e-value, query length, and identity percentage) from three template models present in BiGG (*Escherichia coli* K-12 substr. MG1655, *Klebsiella pneumoniae* subsp. pneumoniae MGH 78578, and *Pseudomonas putida* KT2440). NCBI reference sequence annotation (GenBank) was employed in GPR associations. The RAVEN and COBRA toolboxes for MATLAB were employed in the reconstruction, refinement, and validation of the model. The resulting optimized draft model and constituents of the BOF were manually curated. Protein, RNA, and DNA components of the BOF were estimated based on the total coding sequences. Disconnected metabolites were linked to the metabolic pathways using bioinformatics databases and experimental evidence. Four detailed metabolic modules were carefully added to the M-model to show specific metabolic capabilities of *Thauera sp*. MZ1T: 1) aromatic compound degradation under aerobic and anaerobic conditions, 2) N metabolism including denitrification (with nitric and nitrous oxide partial denitrification), oxidative phosphorylation with nitrate as electron acceptor, and DNRA, 3) PHAs and PHB production, and 4) EPS precursor production. The resulting model was validated using experimental data retrieved from the literature. The iterative model refinement process included manual curation, gap-filling, and curation under heterotrophic conditions with different oxygen concentrations depending on the experimental environments. PHB and EPS production was simulated using a set of 36 C sources under aerobic and anaerobic conditions to estimate the compounds with higher yields. The final model (*i*Thauera861), containing 1,744 metabolites, 2,384 reactions, and 861 genes, predicted growth up to 95% of accuracy for 60 C and N substrates.

We employed a semi-automated approach to reconstruct the M-model of *Thauera sp*. MZ1T. Semi-automated strategies have been successfully applied to build M-models for various microorganisms [23–27] and specifically for bacteria involved in wastewater treatment [24,25]. An initial draft model of *Thauera sp*. MZ1T was generated based on the functional genome annotation retrieved from the NCBI Reference Sequence database: GCA_000021765.1. For this we selected three well-curated and previously validated template M-models for Gram-negative bacteria from the BiGG database [28]. The templates were *Escherichia coli* K-12 substr. MG1655 (*i*ML1515) [29], *Klebsiella pneumoniae* subsp. pneumoniae MGH 78578 (*i*YL1228) [30], and *Pseudomonas putida* KT2440 (*i*JN746) [31]. Protein homology analysis utilizing BLASTp from the RAVEN Toolbox [32] between *Thauera sp*. MZ1T and the three reference models were performed to determine common metabolic resources, i.e. metabolites, reactions, and genes (Fig 1) [24–26,33–36].

We developed a semi-automated reconstruction strategy using three optimal sets of parameters for BLASTp. A set of 391 reactions and their GPR associations were manually curated to use as a quality control check of the GPR associations for each template model (S1 Material). These final BLASTp parameter criteria were applied to the reconstruction for each template model: 1) *Escherichia coli* K-12 substr. MG1655 (*i*ML1515) with e-value = 1e-5, query length = 100 aa, and identity percentage = 35%; 2) *Klebsiella pneumoniae* subsp. pneumoniae MGH 78578 (*i*YL1228) with e-value = 1e-5, query length = 100 aa, and identity percentage = 30%; and 3) *Pseudomonas putida* KT2440 (*i*JN746) with e-value = 1e-10, query length = 120 aa, and identity percentage = 40%. These are similar to the BLASTp cutoffs determined in other semi-automated strategies for bacterial metabolic reconstruction [23–26,33]. The draft semi-automated metabolic model from the three template models contained 1,539 metabolites and 1,721 reactions distributed in three cellular compartments (cytoplasm, periplasm, and extracellular space) with a total of 957 genes (including 98 genes from the template models). This draft model showed higher proportions (>20%) of correct gene associations and fewer template genes (<30%) compared to other semi-automatic models built with a unique set of BLASTp parameters [24–27,35].

### Model refinement: Curation and Gap-filling

The resulting draft model required detailed refinement steps in order to capture the metabolic versatility present in *Thauera sp*. MZ1T. The refinement process was performed in four major stages: 1) manual curation of the existing GPR associations in the draft model, 2) removal of metabolites, reactions, and genes only present in the template models, 3) gap-filling of the disconnected metabolites and blocked reactions based on bioinformatics databases and experimental evidence, and 4) addition of new metabolic pathways and GPR associations considering unique metabolic capabilities of *Thauera sp*. MZ1T (See Methods).

In the first stage of the manual curation, each GPR association of the draft model was carefully reviewed employing the relevant bioinformatic databases for metabolic modeling such as BiGG [28], BioCyc [37], KEGG [38], and MetaNetX [39]. A total of 85 reactions were incorrectly added to the draft model based on protein homology, i.e. incorrect genes assigned to a reaction (See Methods). Close to 80% of the reactions removed in this stage were related to transport reactions (outer and inner membrane), another19 reactions related to carbohydrate and lipid metabolism were removed from the model as well. Additionally, 103 existing GPR associations were manually modified, removing 67 *Thauera sp*. MZ1T (TMZ) genes and including 45 new TMZ genes into the M-model. Most of the TMZ genes removed (41 genes) from the model were wrongly assigned to oxidoreductases and transport reactions. Only 10 of the TMZ genes eliminated from the M-model were unique genes among the GPR associations.

The resulting curated model of the first stage was further refined to remove exogenous metabolites, reactions, and genes from the reference organisms that are not part of *Thauera sp*. MZ1T’s metabolism. A total of 98 template genes were identified and labeled from the GPR associations to be removed. From the 98 template genes, 56 TMZ genes were identified and mapped into the GPR associations of the model, affecting a total of 39 reactions. The remaining 42 exogenous genes were eliminated from the GPR associations. The 39 reactions affected belong to amino acid metabolism, carbohydrate degradation, cofactor and prosthetic group biosynthesis, and inorganic compound and ion transport.

After manual verification of GPR associations and template gene removal, disconnected metabolites and reactions of the *Thauera sp*. MZ1T M-model were connected to the metabolic network by employing gap-filling strategies from the COBRA Toolbox [36] using bioinformatics databases as reference (BiGG [28], BioCyc [37], KEGG [38], and MetaNetX [39]) as well as experimental evidence from the literature [1,12,15–17,20]. From the bioinformatics databases, 52 metabolites, 84 reactions, and 39 TMZ genes were included in the GEM to ensure connectivity across metabolites and subsystems. Most of the curated pathways belong to amino acid metabolism, carbohydrate degradation, inorganic and ions transport, cofactor and prosthetic group biosynthesis, oxidative phosphorylation, and the nucleotide salvage pathway. Additionally, over 70 different growth experiments under aerobic and anaerobic conditions were employed to interconnect and add multiple C and N sources to the M-model. Transport reactions involved in the consumption of C and N sources from experimental evidence were associated with TMZ genes using specific transporter information from NCBI [1], PATRIC [40], and TransportDB [41].

Lastly, specific metabolic pathways were added to the M-model to accurately represent specific metabolic capabilities of *Thauera sp*. MZ1T. Four major pathways were added: 1) aromatic compounds degradation, including the aerobic and anaerobic degradation of benzene-related compounds; 2) N metabolism considering denitrification (with nitric and nitrous oxide partial denitrification), oxidative phosphorylation with nitrate as electron acceptor, and DNRA; 3) PHAs and PHB production from multiple C sources, and 4) EPS precursors biosynthesis. A total of 63 metabolites and 116 reactions were successfully included in the GEM distributed in 22 new specific pathways. After the four stages of the model refinement, the number of TMZ genes increased to 861 (Fig 1).

### Model properties

**Fig 2:**
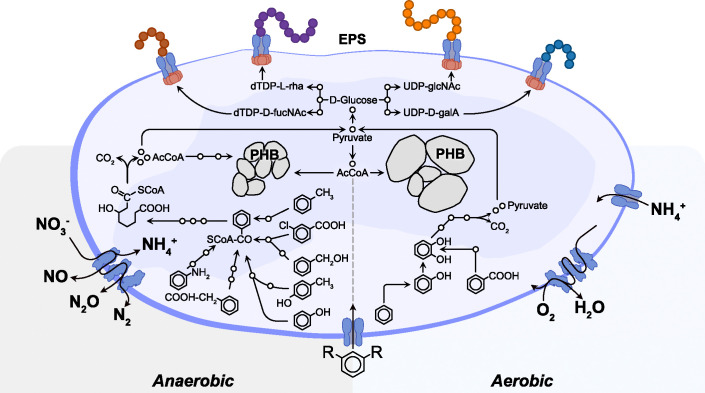
Metabolic properties of *Thauera sp*. MZ1T represented in *i*Thauera861. Anaerobically (left side), *i*Thauera861 deploys six specific pathways to degrade aromatic compounds, as depicted inside the cell diagram (from left to right): 4-chlorobenzoic acid, benzyl alcohol, *p*-cresol, aniline, and phenylacetic acid. These metabolites are converted into a common intermediate, benzoyl-CoA, and finally into acetyl-CoA and pyruvate as the main compounds to connect with global C metabolism. In absence of oxygen, *i*Thauera861 employs nitrate (NO_3_) as the main electron acceptor, converting the NO_3_^-^ into N_2_ through the denitrification pathway, or ammonium (NH_4_^+^) using DNRA. In the presence of oxygen (right side), *i*Thauera861 contains specific enzymes to oxidize aromatic compounds such as benzene, toluene, benzoate, and derivatives and convert them into pyruvate. Oxygen is employed as the key electron acceptor instead of NO_3_^-^. In both conditions, the M-model can produce PHB using acetyl-CoA as the main C precursor, with higher yields of PHB biosynthesis estimated in presence of oxygen. *i*Thauera861 contains six specific EPS biosynthetic reactions using dTDP-D-N-acetylfucosamine (brown), dTDP-L-rhamnose (purple), UDP-D-galactose (blue), and UDP-N-acetylglucosamine (orange) in different proportions.

The final *Thauera sp*. MZ1T metabolic model (*i*Thauera861) consists of 1,744 metabolites, 2,384 reactions, and 861 genes, representing 22% of all annotated coding genes of the genome retrieved from the NCBI GenBank database. Thus, the gene coverage for *i*Thauera861 is in the range of other bacterial metabolic models (15–30%) [24–27,29–31,33]. *i*Thauera861was validated using over 70 experimental growth results under aerobic and anaerobic conditions with multiple C and N sources. *i*Thauera861 contains all metabolites, reactions, and genes involved in aromatic compound degradation, total and partial denitrification, DNRA, PHAs and PHB production, and EPS precursor biosynthesis (Fig 2). The complete distribution of the metabolic pathways present in the M-model is organized into 57 subsystems (including inner, outer, and exchange transport reactions) based on the biological role of the reactions and metabolites involved.

Most of the reactions included in *i*Thauera861 belong to cellular transport (inner, outer, and extracellular reactions) with 26%, amino acids and lipids metabolism (12% each), cofactor, and prosthetic group biosynthesis (10%), cell envelope biosynthesis (7.5%), alternate C metabolism (6.5%), and nucleotide metabolism (including *de novo* synthesis and nucleotide salvage pathways, 6%). Specific metabolic pathways of *Thauera sp*. MZ1T, such as aromatic compounds degradation (2.6%), EPS biosynthesis (1.5%), PHA and PHB production (1.2%), and N metabolism (0.6%) represent less than 6% of the entire metabolism represented in the M-model. The three prokaryotic M-models used as templates during the reconstruction and refinement steps share 1,022 reactions with *i*Thauera861. Over 90% of the reactions present across the four metabolic models (the templates and *i*Thauera861) are related to core bacterial pathways, i.e. amino acids, alternate C metabolism, cofactor metabolism, glycolysis, gluconeogenesis, lipids, nucleotide metabolism, the TCA cycle, and transport reactions. Parts of the aromatic compound degradation, PHAs and PHB production, and N metabolism pathways were extracted from *Escherichia coli* K-12 substr. MG1655 and *Pseudomonas putida* KT2440 models (S2 and S3 Materials).

### Accurate growth and phenotype predictions of *Thauera sp*. MZ1T

*i*Thauera861 was validated employing experimental data retrieved from the literature using several C and N sources under aerobic and anaerobic conditions, capturing most of the metabolic capabilities of *Thauera sp*. MZ1T. Over 70 different experimental growth conditions were examined using amides, amines, amino acids, aromatic compounds, carbohydrates, nucleotides, and organic acids as C sources, in addition to 11 organic (amino acids, amino compounds, and nucleotides) and inorganic (ammonium, nitrate, and nitrite) compounds as N sources to evaluate *i*Thauera861 accuracy [1,12,15–18,20]. Under anaerobic conditions, nitrate was provided as the main electron acceptor instead of oxygen to guarantee energy production. Additionally, PHB and EPS production was estimated through Flux Balance Analysis (FBA) and complementary COBRA Toolbox algorithms under aerobic and anaerobic conditions [36,42]. The maximum yield per C molecule and C:N ratios were calculated *in silico* for PHB and six variants of EPS under aerobic and anaerobic conditions in the steady-state (See Methods). Based on the maximum yields for PHB and EPS production, we identified the main lethal genes for 36 C sources under different oxygen levels.

*Thauera sp*. MZ1T aerobic metabolism has been studied mostly for wastewater or for aqueous environments in the presence of different C and N sources, often with a focus on evaluating its capability of degrading pollutants and producing PHAs and EPS to provide structure and consistency to the microbial community [9,12–16,20,21,43]. Initially, we validated *i*Thauera861 versatility to employ different C sources from aerobic experimental conditions of the literature [12,15–17,20]. Table 1 compares the qualitative experimental results of *Thauera sp*. MZ1T growth utilizing a wide range of C and N sources against the predicted qualitative simulations of *i*Thauera861. We utilized GN2 Biolog plate results from Allen 2002 [16] to test the accuracy of *i*Thauera861 predictions using qualitative data. A total of 46 C sources resulted in significant growth using ammonium as the sole N source under aerobic conditions (See Methods). Six out of the 46 C sources were not mapped to the M-model due to the lack of evidence about the metabolic pathways in *Thauera sp*. MZ1T to consume these metabolites (α-ketovaleric acid, bromo succinic acid, liaconic acid, mono-methyl succinate, Tween 40, and Tween 80). The remaining 40 C sources were used to predict the growth values through FBA with available oxygen, N, phosphorus, and mineral requirements of *Thauera sp*. MZ1T. A detailed list of the C sources evaluated with the experimental and simulation results are provided in S4 Material. From the 40 C sources with significant growth results, *i*Thauera861 successfully predicted significant growth values in 36 of the C sources under aerobic conditions with four false negative estimations (i.e. formic acid, L-alanyl-glycine, L-carnitine, and urocanic acid), and no false positive predictions. An accuracy and sensitivity of 90% with 100% positive predictions were achieved when the M-model was validated with the 40 different C sources. Additionally, the experimental growth values were classified into three categories based on OD600 measurements: high, medium, and low (See Methods). The growth rates of the 36 C sources were distributed in nine high, 16 medium, and 11 low measurements. The growth values predicted were normalized and sorted following the same three categories from the experimental measurements (high, medium, and low). We found that *i*Thauera861 predictions quantitatively matched 30 out of 36 growth rate value classifications. Two incorrect classifications were performed per category with seven correct predictions out of nine in high growth values, 13 out of 15 in medium growth values, and nine out of 11 in low growth values. The six incorrect quantitative predictions are: cis-aconitic acid experimentally classified as high but predicted as medium (underestimation), α-ketoglutamic acid experimentally classified as high but predicted as low (underestimation), L-serine experimentally classified as medium but predicted as low (underestimation), L-histidine experimentally classified as medium but predicted as high (overestimation), L-phenylalanine experimentally classified as low but predicted as medium (overestimation), and L-threonine experimentally classified as low but predicted as medium (overestimation).

**Table 1:** Summary of the growth rates evaluated using different carbon and nitrogen sources under aerobic and anaerobic conditions.

Carbon sources	Nitrogen sources	oxygen	true positive	false negative	total conditions	reference
organic acids	NH_4_^+^	+	12	0	12	[16,17]
carbohydrates	NH_4_^+^	+	1	0	1	[16,17]
alcohol derivatives	NH_4_^+^	+	2	0	2	[16,17]
amino acid and derivatives	NH_4_^+^	+	15	1	16	[16,17]
nucleotide derivatives	NH_4_^+^	+	3	0	3	[16,17]
amines and amides	NH_4_^+^	+	3	0	3	[16,17]
others	NH_4_^+^	+	0	3	3	[16,17]
acetate	organic and inorganic	+	14	0	14	[12,15–17,20]
aromatic compounds	NH_4_^+^	+	11	1	12	[15,16,44]
aromatic compounds	NH_4_^+^ and NO_3_^-^	-	7	2	9	[15,16,44]

Next, the distributions of reaction fluxes across the 57 subsystems in each of the 36 conditions were estimated and clustered to compare the subsystem activity per condition. 49 of the 57 subsystems were active at least in one of the GN2 Biolog plate simulations. The subsystem flux values were z-score normalized across all the experimental conditions to highlight the flux activity changes (S5 Material). The flux distributions of the subsystems show three main C source clusters: 1) high normalized flux activity values in most of the subsystems, including 2,3- butanediol, citric acid, D-gluconic acid, D-glucose, gulonic acid, inosine, uridine, and thymidine; 2) medium normalized flux activity values across the subsystems comprehending α-ketoglutaric acid, β-hydroxybutyric acid, cis-aconitic acid, D-alanine, γ-amino butyric acid, glycerol, L-alanine, L-asparagine, L-aspartic acid, L-glutamic acid, L-glutamine, L-histidine L-leucine, L-proline, L-serine, L-threonine, pyruvic acid, succinic acid, and urocanic acid; and 3) low normalized flux activity values in the majority of the subsystems comprising 2-amino ethanol, acetate, alaninamide, β-hydroxyphenylacetic acid, L-lactic acid, L-ornithine, L-phenylalanine, phenyl ethylamine, propionic acid, and putrescine.

Furthermore, an *in silico* gene essentiality analysis was performed to determine which genes are required for the growth of *Thauera sp*. MZ1T under the 36 conditions identified by the GN2 Biolog plates (see Methods). Of the 861 genes, 578 TMZ genes were labeled as non-relevant genes (67%), 164 genes were included in the growth-reducing genes group (19%), and the remaining 119 genes were determined as lethal genes (14%). Close to 50% of lethal genes are concentrated in GPR associations linked to transport (inner and outer membrane) and cofactor and prosthetic group biosynthesis. Less than 2% of these genes are distributed in alanine and aspartate metabolism, chlorophyll and porphyrin metabolism, murein recycling and tRNA charging. The growth-reducing genes cluster was filtered for genes having a negative effect in more than 5 experimental conditions to determine which of these genes impact certain C source metabolisms.

Nitrogen uptake versatility was validated using inorganic and organic N sources from the literature [12,15–17,20]. The N assimilation and metabolism was qualitatively tested *in silico* employing three inorganic N compounds (ammonium, nitrite, and nitrate) and eleven amino acids as organic N sources, i.e. D-alanine, L-alanine, L-asparagine, L-aspartate, L-glutamate, L-histidine, L-leucine, L-phenylalanine, L-proline, L-serine, and L-threonine. Acetate was utilized as the sole C source for each N condition in the presence of oxygen. *i*Thauera861 accurately predicted the qualitative growth of the experimental results using 14 diverse inorganic and organic N sources (100% of accuracy for the complete set of N sources under aerobic conditions). Furthermore, based on the available transport reactions identified and mapped in the *Thauera sp*. MZ1T M-model, we tested 65 N compounds *in silico* with a predicted gene association to identify potential N sources under aerobic conditions with acetate as the C supply (excluding the 14 experimental growth conditions previously validated). A total of 33 N sources were consumed, distributed in amino acids and derivatives (13 sources), nucleotides and derivatives (12 sources), amides (4 sources), amines (3 sources), and an inorganic N compound (1 source). We estimated which nutrients could be utilized by *Thauera sp*. MZ1T as C and N source when the metabolite is provided without any additional C and N source. We evaluated 73 nutrients based on N sources from the literature experiments and the transport reactions included in the model during the reconstruction process with acetate as the sole C substrate. *i*Thauera861 predicted significant growth using 40 different metabolites (24 amino acids and derivatives, 11 nucleotides and derivatives, 3 amines, and 2 amides) under aerobic conditions.

### Metabolism of aromatic compounds in the presence and absence of oxygen

*Thauera sp*. MZ1T contains highly specialized enzymes involved in the degradation of aromatic compounds under aerobic and anaerobic conditions using organic and inorganic N sources [45–51]. In the presence of oxygen, diverse phenol and benzene-related compounds such as phenol, *p*-cresol, benzoate, 4-hydroxybenzoate and *m*-xylene can be aerobically metabolized to catechol intermediates (catechol, 4-methylcatechol or 3-methylcatechol) [45]. Through the catechol meta-cleavage pathway, the bacterium converts catechol and its derivatives into pyruvate or/and acetyl-CoA, which are core metabolites highly interconnected to the global metabolism of the Gram-negative bacterium (Fig 2). The same metabolic strategy has been identified in other organisms involved in wastewater treatment such as *Pseudomonas putida* and *Rhodocyclaceae* species [45–47]. To validate this strategy in our M-model, we evaluated the performance of *i*Thauera861 on 12 aromatic compounds commonly present in wastewater derived from chemical industries [52,53]. Setting experimental growth conditions as previously done [15,16,44], the M-model was constrained to consume a single aromatic substrate, with ammonium as the sole N source, and all the essential minerals required by *Thauera sp*. MZ1T provided under aerobic conditions (Table 1). *i*Thauera861 predicted growth on 11 out of 12 aromatic compounds in presence of oxygen with 11 true positives (4-hydroxybenzoate, 4-hydroxyphenylacetate, aniline, benzoate, benzyl alcohol, *m*-xylene, *p*-cresol, phenol, phenylacetate, and toluene) and only one false negative prediction (indole). For all true positive scenarios, the mechanism utilized by the M-model for the aerobic catabolism of these aromatic substances involved the catechol meta-cleavage pathway, with 2,3-dioxygenase as the initial key enzyme of this metabolic pathway. Of the 11 true positive predictions, 4-hydroxybenzoate as the sole C source showed the highest growth rate value (0.13 h-1), meanwhile *m*-xylene resulted in the lowest growth rate (0.03 h-1). However, considering the yield per C molecule of each aromatic compound, phenol displayed the highest growth rate per C molecule and *m*-xylene remained as the aromatic compound with the lowest growth rate per C molecule.

Besides the broad metabolic capability to degrade aromatic substrates under aerobic conditions, *Thauera sp*. MZ1T displays the metabolic versatility to consume a wide variety of these metabolites in the absence of oxygen using nitrate as the final electron acceptor [49–51]. Instead of the catechol meta-cleavage pathway utilized under aerobic conditions, this bacterium employs highly specialized metabolic strategies to consume aromatic substrates anaerobically and transform them into benzoyl-CoA as common intermediate [48,50]. In further downstream metabolic steps, benzoyl-CoA is catabolized by hydrolases and oxidoreductases to three molecules of acetyl-CoA, a metabolite that plays a key role as an intermediate for several subsystems of *Thauera sp*. MZ1T [1,12,50]. Similar anaerobic mechanisms have been observed in well-studied denitrifying bacteria such as *Rhodopseudomonas palustris* and *Azoarcus envasii* [48,50]. We implemented a total of 14 metabolites, 21 reactions, and 8 genes in the M-model allocated to the aromatic compound metabolism subsystem for the anaerobic degradation of the aromatic substrates (Fig 2). Using nine anaerobic experimental conditions from the literature [15,16,44], *i*Thauera861 was also validated with nine aromatic compounds under anaerobic conditions, replacing oxygen with nitrate as the final electron acceptor (Table 1). The model predicted seven (4-hydroxybenzoate, benzoate, benzyl alcohol, *p*-cresol, phenol, phenylacetate, and toluene) out of nine aromatic compound utilization in the absence of oxygen and two false negatives (4-aminobenzoate and aniline). Of the seven true positive predictions, *p*-cresol resulted in the highest growth rate (0.17 h-1), meanwhile phenylacetate showed the lowest growth rate (0.09 h-1). Regarding the C effect per molecule of each aromatic compound to the growth rate, phenol stood as the highest growth rate per C molecule, while phenylacetate remained as the aromatic compound with the lowest growth rate per C molecule.

**Fig 3:**
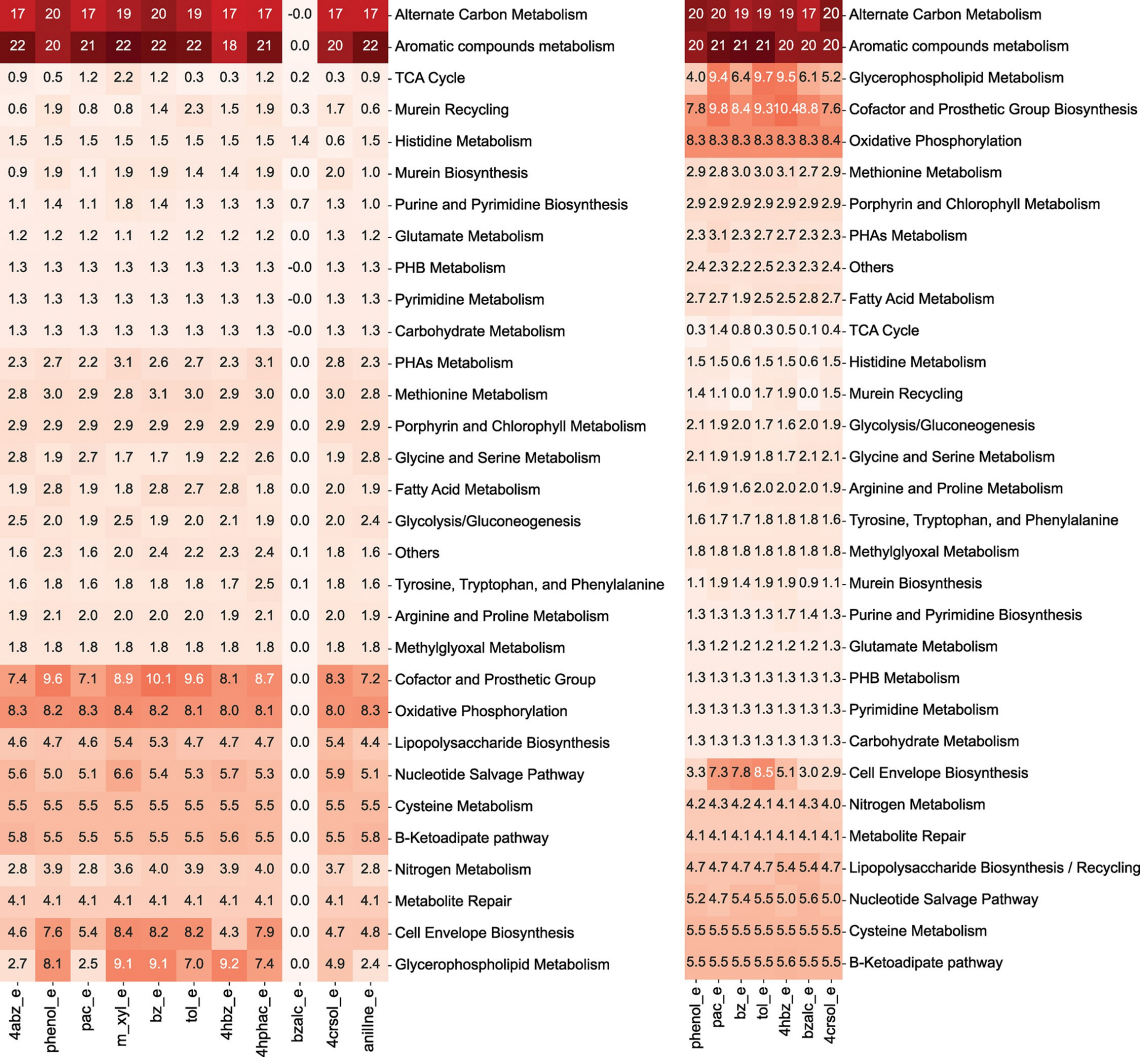
Heatmap containing the -log10 transformation of the Mann Whitney p-values for the up-regulated subsystems using sampling results of the optimal growth values under aerobic and anaerobic conditions. The flux distributions for each aromatic C source were estimated through sampling analysis and the resulting values were grouped and averaged per subsystem. Each growth condition was compared against acetate to determine the upregulated and downregulated subsystems through the Mann Whitney U test. The p-values were plotted using -log10 transformation to identify which metabolic pathways were significantly upregulated. The eleven aerobic experimental growth conditions are displayed on the left and the seven anaerobic denitrifying conditions are shown on the right. Abbreviations: 4abz_e, 4-aminobenzoate; 4crsol_e, *p*-cresol; 4hbz_e, 4-hydroxybenzoate; 4hphac_e, 4-hydroxyphenylacetate; anilIne_e, aniline; bz_e, benzoate; bzalc_e, benzyl alcohol; m_xyl_e, *m*-xylene; pac_e, phenylacetic acid; phenol_e, phenol; tol_e, toluene.

Furthermore, we determined the variation of metabolic fluxes across different subsystems in *i*Thauera861 through sampling analysis using the true positive predictions of the aromatic compounds under aerobic (11) and anaerobic (7) conditions (See Methods). We identified the upregulated and downregulated subsystems per aromatic C source using the p-values of the Mann Whitney U test with acetate as substrate reference (Fig 3). Under aerobic conditions, 10 out of the 11 growth conditions upregulated more than 50% of the subsystems, while benzyl-alcohol as substrate downregulated most of the systems. The higher values in the growth rates and significant upregulation in several pathways supports *Thauera* sp MZ1T’s higher efficiency in using aromatic compounds as C substrates compared to acetate, which permits an increase in biosynthesis of amino acids, lipids, nucleotides, and secondary metabolites.

In both aerobic and anaerobic conditions, the aromatic compounds and alternate C metabolisms presented the highest upregulation values, followed by cofactor and prosthetic group biosynthesis, oxidative phosphorylation, glycerophospholipid metabolism, and cell envelope biosynthesis. Core metabolic pathways such as amino acid subsystems (arginine, glutamate, glycine, histidine, phenylalanine, proline, serine, tryptophan, and tyrosine metabolism), TCA cycle, glycolysis/gluconeogenesis, porphyrin and chlorophyll metabolism, carbohydrate, and fatty acid metabolisms showed low upregulation values with p-values between 1e-1 to 1e-3. Specific subsystems such as cysteine and methionine metabolism of the amino acids, B-ketoadipate pathway, lipopolysaccharides biosynthesis, nucleotide salvage pathway, and metabolite repair were upregulated with p-values between 1e-3 to 1e-5. The N metabolism was significantly upregulated in anoxic conditions compared to the oxic scenarios, which is due to high metabolic activity of the denitrification pathway in absence of oxygen.

### PHB and EPS optimization under aerobic conditions

*Thauera sp*. MZ1T is capable of producing multiple polymers depending on the resources available and the environmental conditions. The most relevant and best-studied polymers produced by this microorganism are PHB and EPS [8,9,12,15–17,20,21]. PHB accumulation occurs in the cytosol of the bacterium under high C:N ratios and different oxygen concentrations, allowing the microorganism to store nutrients inside the cell. EPS biosynthesis in *Thauera sp*. MZ1T requires four main precursors in different proportions that work as the scaffold for these polymers, combined with carbohydrates or/and lipids [15–17,20,54,55]. Depending on the proportion of the main precursors and the content of oligosaccharides and lipids in the structure, the EPS can significantly change its structural properties directly impacting floc formation and permeability of the nutrients in the environment [1,15–17,20]. Currently, there is limited understanding of the effect of EPS concentration on floc formation and wastewater treatment. Additionally, the mechanisms, metabolic strategies, and environmental conditions required for synthesizing and optimizing production of PHB and EPS in *Thauera sp*. MZ1T are not well understood. Thus, we exploited the prediction capabilities of *i*Thauera861 to determine the impact of C source, C:N ratio, oxygen presence, and GPR associations in the production of PHB and six different variants of EPS.

First, we evaluated *i*Thauera861 precision to predict PHB production using Colpa 2020 experiments under aerobic N-limiting conditions [12]. We used acetate as the sole C and ammonium as N source with the minerals and micronutrients required for *Thauera sp*. MZ1T growth (See Methods). Different acetate:ammonium ratios (a sensitivity analysis from 1:1 to 20:1 C:N ratios) were evaluated to determine the optimal C:N proportion for PHB production. Colpa in 2020 tested 10 g/L of acetate in a 10:1 C:N proportion (mol/mol) using ammonium as the sole N source with PHB production resulting in 50% of cell dry mass. Our results showed that C:N proportions above 5:1 using acetate and ammonium as the C and N sources allowed PHB production without compromising the growth rate of *Thauera sp*. MZ1T. Lower C:N ratios affected the growth rate or impacted the N consumption fluxes, since higher PHB production rates directly affect the biomass formation and thus the N requirements. However, *i*Thauera861 does not provide optimal solutions for the maximum PHB production rate for higher C:N ratios. Above 5:1 C:N ratios, C consumption is redirected to the PHB production, which is only limited by the biomass growth rate. Higher C:N proportions in metabolic modeling without experimental constraints led to increasing PHB production rates. PHB biosynthesis is usually affected by multiple parameters outside of the scope of metabolic modeling, such as temperature, pH, regulated genes, and protein-cost production [56–58].

**Fig 4:**
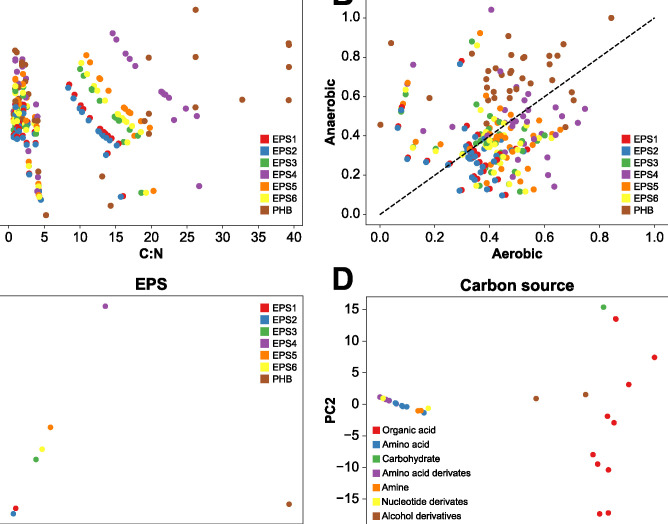
Effect of the C:N ratio and different carbon sources on production of PHB and EPS. **A** Scatter plot depicting the production per C molecule and C:N ratios of PHB and EPS under aerobic condition with 36 C sources. No clear tendency can be observed from 0 to 5 using the C:N ratio as reference. With C:N ratio above 5:1 a clear increase in the yield is noticed showing the highest yields when C:N ratios are higher for PHB production. **B** Comparison of the yields per C molecule in the presence and absence of oxygen. Most of the yields are concentrated in the center of the plot, meaning that the yields are remarkably similar independently of the oxygen concentration. **C** PCA plot displaying the two first component scores of the C:N ratios considering the PCA coefficients of PHB and EPS under aerobic conditions. The PCA represents 99% of the variability of the data. Two main clusters can be identified, the cluster of the EPS (left side) and the cluster of the PHB (right side). **D** First two component scores of the C:N ratio PCA under aerobic conditions, considering the PCA coefficients of C sources. The compounds were grouped by their common functional groups. Interestingly, two main clusters were recognized, the common functional groups with N (left) and the compounds without N in their chemical composition (right).

*i*Thauera861 was further exploited to identify the effect of different C sources and C:N ratios under aerobic and anaerobic conditions on the production of PHB and six specific EPSs (see Methods) [57,59,60,61]. When oxygen is available, the highest production for the seven polymers was obtained using inosine as the sole C source. Nucleotide compounds such as inosine, thymidine, and uridine, performed as the most efficient C substrates to produce PHB and the six variants of EPS. Other substrates such as alcohol derivatives, amides, amines, amino acid derivatives, amino acids, and organic acids, generated lower yields across all six EPS molecules except for PHB. Specifically, organic acids (acetate, propionate, and pyruvate) performed on average as the least efficient C substrates to produce PHB and EPSs. Despite the poor productivity of polymers from organic acids, the lowest production value was achieved by the amine phenethylamine with less than the sixth part of the efficiency obtained with the nucleotide compounds. Based on the nutrients requirements to achieve the highest yield values under the established growth rate and polymer contributions, the C:N ratios per condition were estimated based on C and N uptake fluxes (Fig 4A). The highest C:N ratios were achieved with the C derived from organic acids, carbohydrates, and alcohol derivatives (compounds without N) across the seven different polymers. Growth conditions using substrates containing N such as amides, amines, amino acid derivatives, amino acids, nucleotide derivatives, exhibited lower C:N ratio values. For C:N ratio values between 0 to 5 under aerobic conditions, the polymers presented a similar outcome generating a unique cluster of all the yields. A different trend was observed for C:N ratios in the range of 10–25, where the EPSs were distributed in three main clusters: EPS1 and EPS2 located in the C:N ratios range of 10–15, EPS 3, EPS5, and EPS6 conformed the second cluster in the C:N ratios range of 15–20, and EPS4 as the only member of the third cluster (range of 20–25). The PHB yield values had two clear dispositions, with a random distribution from the C:N ratios range of 0–5 and a gradual increase of the yields for C:N ratios above 5, reaching the peak of the yield values at a C:N ratio of 25. C:N ratios for PHB production above 25 did not increase the yield values from the C:N ratios range of 20–25. This PHB production’s strong dependency to the C:N ratio compared to the EPS biosynthesis requirements has been extensively reported for different bacteria mainly under aerobic conditions [22,62–64]. However, no clusters were identified when the yield distributions were analyzed considering the C:N ratio values across the different polymers under anaerobic conditions (S6 Material). EPS3, EPS4, and PHB achieved greater yield values under aerobic conditions, meanwhile the rest of the EPSs distributed evenly independent of the oxygen presence. A principal component analysis (PCA) was carried out to determine the impact of the C:N ratios on the polymers and various C substrates (Fig 4C and 4D). Two main clusters were recognized when the first two PCA scores of the C:N ratios are displayed and grouped by polymer, with the first cluster conceived by the EPS compounds (left side) and PHB as a sole element of the second cluster (right side). A similar trend is exhibited in the second PCA of the C:N ratios grouped by substrate group; the cluster of substrates with N in their chemical composition, i.e. amides, amines, amino acid derivatives, amino acids, and nucleotide derivatives showed a substantially different pattern in the C:N ratio (left side) compared to the C compounds without N such as alcohol derivatives, carbohydrates, and organic acids (right side). The presence of N in the substrates from the first cluster reduced the requirements of an external N supply like ammonium compared to the second cluster of C compounds. However, the ammonium requirement as a N supply positive correlates to higher production rates for the second cluster of C compounds.

**Fig 5:**
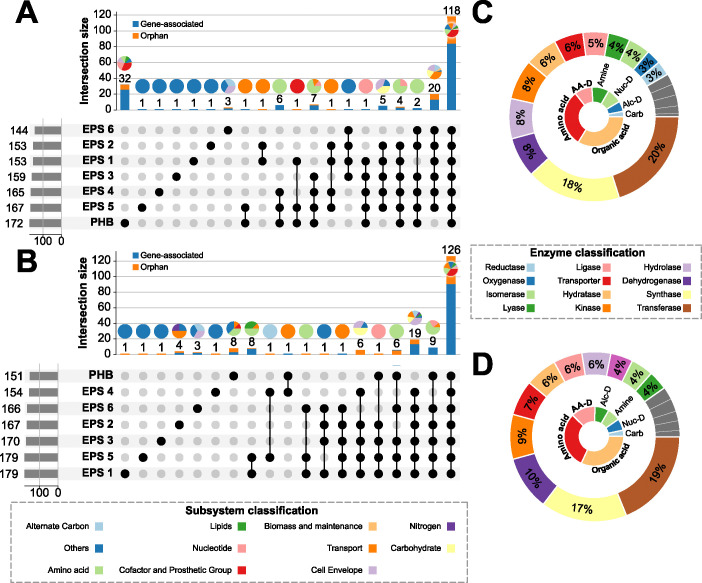
Reaction essentiality analysis for the production of PHB and EPSs under aerobic and anaerobic conditions using different carbon sources. **A** Upset plot with 20 distinct groups of lethal reactions for PHB and six EPSs under aerobic conditions. Pie charts of the reaction subsystem distribution above each bar from the lethal reaction groups. **B** Upset plot with 19 diverse groups of lethal reactions for PHB and six EPSs under anaerobic conditions. Pie charts of the reaction subsystem distribution above each bar from the lethal reaction groups. **C** Percentage distribution of enzyme classification of lethal genes estimated through single gene deletion under aerobic condition (outer plot) and the C sources affected by the lethal reactions (inner plot). **D** Percentage distribution of the enzyme classification of the lethal genes estimated through single gene deletion under anaerobic condition (outer plot) and the C sources affected by the lethal reactions (inner plot).

Besides the impact of the C:N ratio to the PHB and EPS production efficiency, there has been an interest in the reactions and genes involved in the production and efficiency of PHB and EPS in *Thauera* microorganisms [1,12,15–17,20]. For PHB production, acetyl-CoA acetyltransferase, acetoacetyl-CoA reductase, and PHA synthase were identified as key enzymes in the biosynthesis of this polymer, reducing and even in some environments stopping the PHB accumulation and leading to the death of the bacterium [9,12,16,17]. In *Thauera sp*. MZ1T, mutants have been generated deleting genes involved in the generation of the EPS precursors, polymerization, and excretion resulting in alterations with negative effects on the cell surface organization, intercellular interactions, and floc-forming capacity [16,17]. Some of the mutants generated significantly lower growth rates and EPS biosynthesis, and even in specific scenarios, leading to lethal outcomes. In addition to identifying the impact of C substrate, oxygen, and C:N ratio on the production of polymers in *Thauera sp*. MZ1T, we utilize *i*Thauera861 to evaluate on a genome-scale level the impact of the reactions and genes to the PHB and EPS production. A reaction essentially analysis was executed to determine the effect of each reaction and its GPR association on the growth rate using the experimental conditions of GN2 Biolog plates in two oxygen scenarios (presence and absence). A total of 2,175 reactions were evaluated in the single-reaction deletion analysis with almost 28% of the reactions labeled as orphan reactions (excluding pseudo reactions of the GEM). The lethal reactions were classified considering three main categories: C source, polymer produced, and oxygen presence (See Methods). Of the 2,175 reactions, 208 reactions were labeled as lethal reactions in at least one polymer-producing condition under aerobic conditions (Fig 5A). Close to 75% of the lethal reactions were associated to at least one TMZ gene mostly distributed in transferases (20%), synthases (18%), dehydrogenases (8%), hydrolases (8%), transporters (8%), and hydratases (6%) (S7 Material). 24 of the aerobic lethal reactions were not found in the list of anaerobic lethal reactions, which are directly involved in the oxygen utilization, cofactor and prosthetic group biosynthesis, purine and pyrimidine biosynthesis, cell envelope biosynthesis, and transport reactions across the three compartments (Fig 5C). 118 lethal reactions in oxygen presence affected the seven different polymer growth conditions, mainly impacting the nucleotide, amino acid, cell envelope and other metabolic pathways (including the reactions involved in the production of the PHB and EPS precursors). The growth conditions with PHB production had more unique lethal reactions (32) compared to the entire set of growth conditions for the six diverse variants of EPS (20). Of the 208 lethal reactions under aerobic conditions, the PHB cluster presented the highest number of lethal reactions with 172, while the EPS6 had the lowest number of lethal reactions (144). Almost 80% of the lethal reactions occurred in amino acid derivatives, amino acids, and organic acids as carbon sources.

The number of lethal reactions decreased to 199 under anaerobic conditions (28% of which are orphan reactions) with significant changes in the subsystems affected. Only 15 of the lethal reactions in absence of oxygen were not found in the list of aerobic lethal reactions. However, the metabolic pathways impacted were linked to denitrification, N metabolism, folate metabolism, N transport, and a few of them to the transport across the cellular compartments. The growth conditions for EPS production showed a significantly higher number of lethal reactions (19) compared to the PHB cluster (8). The subsystems distribution and enzymatic classification remained similar in both aerobic and anaerobic reactions, with also 80% of the lethal reactions occurring in amino acids, amino acids, derivatives, and organic acids as C sources when oxygen is absent.

## 3. Discussion

We have reconstructed a comprehensive GEM for an important floc-forming and denitrifying wastewater bacterium using semiautomatic strategies. The high-quality, manually refined, and validated metabolic model of *Thauera sp*. MZ1T unravels the metabolic capabilities of *Thauera sp*. MZ1T under aerobic and anaerobic conditions. Initially, the M-model was reconstructed based on three Gram-negative bacterial reference models from BiGG [28] selected according to their metabolic and physiological similarities (*Escherichia coli* K-12 substr. MG1655 [29], *Klebsiella pneumoniae* subsp. pneumoniae MGH 78578 [30], and *Pseudomonas putida* KT2440 [31]). Unlike other semi-automatic reconstruction approaches for metabolic modeling, we employed a reconstruction strategy considering multiple BLASTp optimal parameters depending on the number of template models. There is no clear consensus on the optimal BLASTp criteria in the metabolic reconstruction process since the BLASTp parameter values directly depend on how similar the studied microorganisms are to the reference models. However, multiple studies have reported BLASTp cutoffs of e-value between 1e-15 and 1e-5, query length of 50 to 150 amino acids, and identity percentage of 20–40% for bacteria [24–27], and even for eukaryotic organisms [35]. Selecting a unique set of BLASTp parameter values can significantly impact the number of false positive (wrong gene assignments) and false negative (missing genes) calls in the GPR associations. In this study, we reduced the number of wrong gene assignments by more than 20%, directly impacting the time required for manual refinement. Additionally, having multiple BLASTp parameters reduced the number of genes from the reference models by almost 30%. While the chosen parameters provided sufficient results for the model of *Thauera sp*. MZ1T, further analyses will be necessary to identify the accuracy of multiple BLASTp parameters optimization for other organisms and template models.

*i*Thauera861 contains close to 22% of the annotated proteins assigned to GPR associations. Compared to the metabolic genes of the template models, *i*Thauera861 only contains a higher percentage of metabolic genes compared to *i*JN746 (14%). However, the updated version of *Pseudomonas putida* KT2440 M-model, *i*JN1463 (27%), as well as *i*ML1515 (31%) and *i*YL1228 (25%) display greater percentages of metabolic genes. Considering the updated template model, the average percentage of metabolic genes in the reference organisms surpasses the percentage in *Thauera sp*. MZ1T by almost 6%. *i*JN1463 and *i*ML1515 include over 400 reactions more than *i*Thauera861, meanwhile *i*YL1228 contains 122 less reactions (S2 Material). The difference among metabolic genes could be related to the lower amount of orphan reactions identified in the template models (20% compared to almost 28% in *i*Thauera861) and the large number of reactions with less than three TMZ genes in the GPR associations (almost 50% excluding the pseudo reactions of the M-model). Further analysis must be performed to determine the metabolic function of multiple hypothetical and putative proteins from the *Thauera sp*. MZ1T annotation to incorporate the new findings in the GPR associations of the model. In addition, we compared the metabolic features of *i*Thauera861 to three extensively validated M-Models of Gram-negative bacteria, i.e. *Azotobacter vinelandii* DJ, *i*DT1278 [26]; *Nitrosomonas europaea* ATCC19718, *i*GC535 [24]; and *Rhodopseudomonas palustris* Bis A53, *i*DT1294 [25], which are involved in the degradation of aromatic compounds, play a role in nitrogen metabolism, PHB production, or are present in wastewater or soil environments. *i*Thauera861 contains a similar number of reactions compared to the *A*. *vinelandii* model *i*DT1278 (2,432), sharing the core metabolic pathways for aerobic metabolism and a similar metabolic mechanism regarding PHB production. *R*. *palustris* Bis A53’s metabolic model, which contains 2,123 metabolites and 2,721 reactions, surpasses *i*Thauera861 by almost 350 metabolites and 400 reactions. However, the *Thauera sp*. MZ1T M-model shares multiple subsystems and metabolic features with *i*DT1294. Both M-models encompass specific reactions for the degradation of multiple aromatic compounds in the presence and absence of oxygen, denitrification, PHB production, and the capability to consume a wide range of C and N substrates. The significant difference in model size is related to *R*. *palustris’* capability to perform anoxygenic photosynthesis, nitrogen fixation, and to grow under four metabolic states (chemoautotrophy, chemoheterotrophy, photoautotrophy, and photoheterotrophy). *i*Thauera861 can grow under aerobic and anaerobic conditions, but solely heterotrophically. The largest difference in the quantity of metabolites and reactions was observed when *i*Thauera861 was compared against *i*GC535 (*N*. *europaea*). *i*GC535 contains only 1,114 metabolites (36% less than *i*Thauera861) and 1,149 reactions (52% less). Additionally, *N*. *europaea* is capable of growing only on a few C and N substrates under strict aerobic conditions [24]. Despite the low metabolic similarities between *N*. *europaea* and *Thauera sp*. MZ1T, these microorganisms can be found in wastewater environments as part of a microbial community, interacting with other bacteria to remove most of the C and N pollutants [65]. *Thauera* organisms play a key role in wastewater treatment contaminated with aromatic compounds, removing these toxic compounds and thus enabling growth of *N*. *europaea* and in turn guaranteeing nitrification [12,16,17,24,56]. Different studies have reported a negative impact of aromatic compounds such as benzene and toluene, on *N*. *europaea*. These aromatics inhibit ammonium oxidation in *N*. *europaea* and trigger an energy drain [66,67].

Our GEM encompasses well-detailed metabolic pathways for degradation of aromatic compounds, both under aerobic and anaerobic conditions. *i*Thauera861 comprehends the consumption of aromatics through the catechol meta-cleavage pathway in the presence of oxygen and the benzoyl degradation pathway when oxygen is not available (S8 Material). This M-model contains more aromatic degradation pathways and metabolic mechanisms than other well-curated models such as *P*. *putida* KT2440 (*i*JN746 and *i*JN1463) [31], *R*. *palustris* Bis A53 (*i*DT1294) [25], *N*. *europaea* ATCC19718 (*i*GC535) [24], or *Geobacter metallireducens* GS-15 (*i*AF987) [33]. *i*Thauera861 shares most of the aerobic aromatic degradation with *i*JN1463, i.e. transformation of most aromatic metabolites into catechol and further catabolization to pyruvate. *i*JN1463 exclusively contains the metabolic pathways for the aerobic consumption of 2,4,6-trinitrotoluene, *o*-xylene, *p-*xylene, vanillin, and vanillate. However, *i*Thauera861 is the first M-model that includes the metabolic pathway for the degradation of aniline in presence of oxygen. Under anaerobic conditions, *i*Thauera861 employs the benzoyl degradation pathway, also found in *i*DT1294 and *i*AF987. However, the specific mechanism to catabolize benzoyl-CoA into acetyl-CoA utilizing the exact same enzymes is shared between *Thauera sp*. MZ1T and *G*. *metallireducens* GS-15 [48]. *i*DT1294 utilizes a slightly different metabolic pathway still channeling metabolites into benzoyl-CoA and producing acetyl-CoA but using different enzymes and intermediates. A similar metabolic strategy has also been characterized for *Azoarcus sp*. CIB [48]. Among all these M-models, *i*Thauera861 stands as the unique metabolic model capable of degrading multiple aromatic compounds under aerobic and anaerobic conditions. Thus, the model developed in this study enables the study of aromatic compound metabolism under various oxygen concentrations, often encountered in wastewater systems.

*Thauera sp*. MZ1T’s metabolic model was carefully employed to identify the impact of different biological parameters involved in the production of PHB and EPSs. Three parameters (C substrate, oxygen presence, and C:N ratio) were shown to have a significant effect on the yield of these polymers. For example, nucleotides as C sources significantly increased the production of PHB and EPS, while substrates lacking N in their chemical structure such as alcohols, carbohydrates, and organic acids, contributed significantly to polymer production. Our results match with experimental results reported previously that found a positive correlation of C substrates without N to the production of PHB and EPS under aerobic conditions [54,60,61,68]. Additionally, *i*Thauera861 predicted the C:N ratio to have a greater impact on the PHB production independently of the C source employed in presence of oxygen than on the other polymers produced. This has been observed previously under different experimental conditions, where the C:N ratio impacts PHB production by *Thauera* sp MZ1T independent of its EPS synthesis [12,16,17,56,57]. Different studies have linked this phenomenon to biological parameters, since PHB biosynthesis is triggered by N-limiting conditions to generate C storage compounds, whereas EPS production is not dependent on the available N substrate concentrations [12,16,17,56,57]. Oxygen appears to have a lesser influence on polymer production than C source and C:N ratio. Under aerobic conditions, *i*Thauera861 only predicted an increase in the production of PHB, EPS3, and EPS4. Our modeling analysis was limited to predict the biosynthesis of only six EPS variants of the wide spectrum of EPS compositions. Many diverse EPS compositions and varying yields have been reported for *Thauera sp*. MZ1T, which results in different structures that influence flocculation [16,17,20]. To date, there is not enough data to conclusively determine which specific EPS variants and C substrates have an influence on the consistency of bacterial flocs [16,17,20]. Deciphering the impact of the main metabolic parameters evaluated in the current study to produce PHB and EPSs can potentially play a role to understand the development and quality of the wastewater microbial community biofilms and granular sludges. The variations in PHB and EPS production identified in our study have meaningful implications for wastewater treatment and bioremediation. Specifically, the composition and concentration of PHB and EPS directly impact the quality of wastewater sludge granules by affecting both the physical integrity and metabolic capabilities of the microbial community. The production of PHB and specific EPS variants aids in flocculation, enhancing the settling and compactness of biomass, which is essential for efficient wastewater treatment. Additionally, these polymers contribute to sludge permeability, which could facilitate nutrient transfer and removal efficiency. This structural support provided by PHB and EPS improves sludge stability and potentially enables more efficient operation of treatment facilities, especially under conditions where nutrient availability fluctuates. Thus, optimizing conditions to enhance PHB and EPS production could offer valuable strategies for improving the quality and performance of wastewater treatment systems.

The GEM reconstructed, refined, and validated in the present study (*i*Thauera861) provides new insights into how key parameters, including C source, oxygen levels, and C:N ratio, influence PHB and EPS production, both essential biopolymers for floc formation in wastewater treatment. Notably, *i*Thauera861 confirms a positive correlation between N-deficient carbon sources and enhanced polymer synthesis, which aligns with experimental data. Our findings also demonstrate that while oxygen presence has limited impact, the C:N ratio plays a dominant role in PHB yield. With applications extending to wastewater treatment, *i*Thauera861 offers a predictive tool for enhancing microbial granule quality by optimizing PHB and EPS production to improve sludge granule compactness, settling, and permeability. This metabolic model thus advances our understanding of *Thauera sp*. *MZ1T* and its role in wastewater treatment systems, providing a foundation for further environmental and bioremediation applications.

## 4. Methods

### 4.1 Draft model generation

The genomic sequence of *Thauera sp*. *MZ1T* was obtained from the NCBI Reference Sequence database [1] (RefSeq code: GCA_000021765.1; genome size: 4.5 Mbp, GC content: 68.3%, and 3,941 proteins). Using the COBRA and RAVEN Toolboxes [32,36] for MATLAB, a draft model was created based on protein homology (BLASTp [69]) to three carefully selected reference models from the BiGG database, chosen to maximize *Thauera sp*. *MZ1T*’s metabolic coverage while minimizing redundant reactions. The template models for *Thauera* sp. MZ1T were selected based on criteria that maximized both the number and quality of homologous GPR associations. The first template was chosen for its highest number of BLASTp hits, prioritizing templates with the best alignment parameters, specifically the lowest e-value and highest query coverage and sequence identity. This ensures that the initial template is most closely related to *Thauera* sp. MZ1T in terms of protein homology and metabolic capabilities. Subsequent templates were selected to add unique GPR associations not present in the first template, allowing inclusion of additional metabolic reactions without redundant overlap. This approach provided a broad, non-redundant foundation for the draft model, aligning closely with *Thauera* sp. MZ1T metabolic profile while maximizing the functional completeness of the GEM. Template models included three Gram-negative prokaryotic organisms: *Escherichia coli* str. K-12 substr. MG1655 (iML1515) [29], *Klebsiella pneumoniae* subsp. pneumoniae MGH 78578 (iYL1228) [30], and *Pseudomonas putida* KT2440 (iJN746) [31]. We performed a sensitivity analysis on BLASTp parameters (e-value, query length, and identity percentage) across 144 combinations, optimizing for the best matches to *Thauera sp*. *MZ1T* while minimizing false-positive GPR associations. In the context of semi-automated reconstruction process for GEM, true positive calls consist on genes correctly assigned in a GPR association, meanwhile a true negative refers to the genes correctly unassigned to a specific GPR association. This included evaluating four e-values (1e-30, 1e-20, 1e-10, and 1e-5), six query lengths (50–200 amino acids), and six identity percentages (20–50%). We employed a set of manually curated reactions and their GPR associations to determine the true positive and negative calls, as well as the false positive and negative predictions. The curated reactions were selected based on the EC numbers identified in the genome annotation and subsequently mapped to BiGG identifiers. Three optimal parameter sets emerged, yielding high-accuracy homology matches and tailored GPR associations for each template model. Redundant reactions from multiple templates were reconciled by selecting those with the highest BLASTp scores, producing a foundational draft model closely aligned with *Thauera* sp. MZ1T metabolism.

### 4.2 Model refinement

Model refinement of the draft model was performed in two steps of manual curation and two gap-filling stages: 1) manual curation of the GPR associations in the draft model added during the initial reconstruction process, 2) removal of metabolites, reactions, and genes only present in the three template models, 3) gap-filling of the disconnected metabolites and blocked reactions based on bioinformatics databases and experimental evidence, and 4) incorporation of new subsystems and GPR associations considering specific metabolic mechanisms of *Thauera sp*. MZ1T.

#### 4.2.1 Manual curation

In the first step of the manual curation, we identified the reactions with exogenous proteins from the template models in the GPR associations in the initial draft model. GPR associations with mixed TMZ and exogenous proteins were modified by removing the exogenous elements from the model. Reactions with only exogenous proteins or enzymatic complexes with exogenous proteins participating in the GPR associations were mapped in *Thauera sp*. MZ1T metabolism using data from the most relevant bioinformatics databases (BiGG [28], BioCyc [37], KEGG [38], and MetaNetX [39]). Exogenous proteins incorporated during the reconstruction phase were replaced with homologous TMZ sequences, utilizing their Enzyme Commission (EC) numbers to reference and confirm their metabolic functions. Additionally, reactions lacking any TMZ proteins in the GPR associations were analyzed through the BLASTp algorithm. Candidate homologous proteins from the *Thauera sp*. MZ1T annotation were identified using BLASTp, comparing them against template proteins assigned to GPR associations for the reaction in other metabolic models of microorganisms listed in the BiGG database. The BLASTp cutoffs were an e-value ≤ 1e-10, query coverage ≥ 75% and identity percentage ≥ 35%. We carried out a second step of manual curation to corroborate the accurate assignment of the GPR associations. The proteins corresponding to each reaction in the partially curated draft model were carefully reviewed, considering the type of metabolic reaction, protein function, and cellular compartment. Protein complexes were accurately adjusted from the GPR associations of the template organisms to match the specific conformations of protein complexes in *Thauera sp*. MZ1T. All validated metabolites, reactions, and GPR associations were allocated in three different cellular compartments (periplasm, cytoplasm, and extracellular compartment). Transport reactions were added using the TransportDB database [41]. Metabolite transport between compartments was curated using BLASTp and bioinformatics databases such as KEGG and BioCyc. Metabolites with missing chemical formulas or/and charges were curated using consensus information across KEGG, Metacyc, and PubChem databases. Furthermore, metabolites not included in any reaction from the model were carefully removed ensuring the stability of the S matrix.

#### 4.2.2 Gap filling

After the initial manual curation, gap-filling was completed in two complementary stages: (1) filling gaps in metabolic pathways already present in the manually curated draft model, and (2) integrating new metabolic pathways identified from bioinformatics databases and literature evidence. This gap-filling approach has been successfully applied in previous prokaryotic model reconstruction processes, where it yielded high accuracy in metabolic prediction for microorganisms relevant to wastewater treatment and ecological environments [23–27]. A targeted semi-automated approach was adopted to selectively integrate reactions and metabolites from databases such as BiGG, KEGG, and BioCyc, specifically refined to align with the unique metabolic capabilities and ecological niche of *Thauera* sp. MZ1T.

The first part of the gap analysis was executed to identify which metabolites remained disconnected in the draft M-model and which reactions were missing in the analyzed metabolic subsystems. Initially, we determined the disconnected metabolites through dead-end analysis algorithms from the COBRA Toolbox [36]. The dead-end compounds were categorized based on their disconnection reason (available in one reaction, only as a substrate or as a product). Later, the reactions required to establish suitable connections of dead-end metabolites were identified and added to the model with their corresponding GPR associations using different bioinformatic databases (BiGG [28], BioCyc [37], KEGG [38], and MetaNetX [39]). Gap filling was also employed to interconnect metabolic pathways already present in the model (amino acid metabolism, denitrification, glycolysis, oxidative phosphorylation, etc.). Finally, dead ends were identified through BioCyc and KEGG subsystem modules and *in silico* synthesized using COBRA gap filling procedures. A final examination was carried out to confirm the production of each dead-end compound using the sink reaction strategy designed for the COBRA Toolbox. Subsequently, we implemented a second round of gap filling to connect metabolites from the medium compositions of different experiments under aerobic and anaerobic conditions reported in the literature. Gap filling procedures identified the required reactions for the assimilation and metabolism of over 70 C and N sources under aerobic and anaerobic conditions. Uptake reactions required for the transportation of the substrates across the three compartments were annotated using BioCyc [37], KEGG [38], MetaNetX [39], and transporters identified through experiments retrieved from the literature. The reactions integrated in the gap filling process with no TMZ genes in the GPR associations were annotated as orphan reactions. We validated the production of each metabolite present in the M-model using FBA to verify the predicted internal fluxes of the reactions.

We concluded the gap filling stage by integrating new metabolic subsystems to the refined model using semi-automated strategies. Specifically, C (aerobic and anaerobic degradation of aromatic compounds, PHB and EPS biosynthesis), and N (denitrification, DNRA, and partial denitrification) metabolism and their respective annotation. The new reaction and metabolite identifiers were designated based on the BIGG database standardization; metabolites and reactions with no available information in BiGG were included in the model. The new reactions were assigned using the EC Number information (through BRENDA [70]) and bioinformatic databases (BioCyc, KEGG, MetaNetX). Meanwhile, the new metabolites were integrated based on specialized databases for biochemical compounds (KEGG, MetaCyc, and PubChem). Detailed information for metabolites and reactions (charge, formula, reversibility, direction, etc.) were obtained from well-reviewed biochemical databases (PubChem [71], UniProt [72], ModelSEED [73], KBase [74], and MetaCyc [75]) or from experimental conditions of *Thauera sp*. MZ1T or closely related *Thauera* members such as *Thauera aromatica* or *Thauera aminoaromatica*. The GPR associations of the reactions identified in other *Thauera* members were determined by protein homology to find the equivalent TMZ proteins. For *Thauera sp*. MZ1T-specific features, like pathways for aromatic compound degradation under aerobic and anaerobic conditions, literature information using experimental evidence was employed to build these specific metabolic subsystems. The functionalities of all reactions were validated using FBA through the model to predict *in silico* growth (biomass production). Reactions added in the last stage of gap filling were evaluated by setting specific constraints in the M-model and performing simulations to measure the internal reaction flux distributions. The remaining reactions with exogenous genes in the GPR associations of the model after the gap-filling stage (incorrectly assigned reactions) were identified and analyzed through Flux Balance Analysis (FBA) using the COBRA Toolbox [36,42]. After completing the gap-filling stage, we optimized the model using FBA across 70 different experimental conditions, allowing us to assess reaction activity under varying environmental parameters. We focused on identifying reactions with exogenous genes in their GPR associations that showed no flux under any growth condition. Consistently inactive reactions underwent manual verification against annotation files and bioinformatics databases (KEGG and BioCyc) to confirm the absence of homologous genes in *Thauera sp*. *MZ1T*, establishing a lack of biological relevance. Verified reactions were then removed from the model. In cases where reactions with exogenous genes carried flux, these were retained as orphan reactions, following optimization to ensure functionality across the intended conditions.

#### 4.2.3 Final quality control and quality analysis

A final quality assessment was executed to guarantee correct GPR associations and successfully balanced metabolites and reactions. We employed the COBRA and RAVEN Toolboxes for MATLAB [32,36] to perform *in silico* single gene-deletion simulations (*in silico* gene knockouts) and check if the GPR associations are properly allocated. Later, we balanced all the reactions and assigned charges and chemical formulas to all the metabolites in the models, verifying the consistency of the model through the Mass and Charge Balance simulations of the COBRA Toolbox on the M-model. Unbalanced reactions were fixed by adding the correct formula and charge of each metabolite. Stoichiometric values assigned in every reaction were carefully reviewed and corrected for unbalanced reactions. The final model was analyzed by seeking for ATP, NADH, and NADPH energy cycles which generate free energy in the M-models, by removing media substrates (i.e., exchange reactions lower bounds constraint to zero) and checking that they do not carry out any flux (zero flux). Duplicate metabolites with different identifiers (i.e., redundant metabolites from different template GEMs) were unified to avoid repeated metabolic reactions or pathways.

### 4.3 Constraints and growth simulations

We utilized experimental conditions from the literature to determine specific medium constraints of the M-model in different oxygen concentrations. For all the experimental growth conditions, C and N uptake rates were calculated depending on the values obtained from results in the literature. When oxygen uptake rate was not specified in the experiments, the oxygen exchange flux was set to a maximum uptake rate value of 1000 and limited based on the C and N substrate exchange rates. Under anaerobic conditions, oxygen consumption rate was set to zero (i.e. oxygen lower bound set to zero). The constraints related to mineral requirements were set based on the BOF estimations. Growth and internal fluxes simulations were performed in the COBRA Toolbox for MATLAB using the FBA procedure and Gurobi Optimizer Version 10.0.3 solver (Gurobi Optimization Inc., Houston, Texas). Mineral stoichiometric coefficients in the BOF of the M-model were set utilizing multiple mineral compositions retrieved from the literature. Ammonium was used as the main N source for initial model estimations under aerobic conditions. In absence of oxygen, ammonium was used as the principal N substrate and nitrate was additionally provided to replace oxygen as the main electron acceptor. Acetate was set as the principal carbon source based on experimental reports in different oxygen concentrations. Mineral uptake fluxes were determined based Subsequently, over 70 preliminary experimental growth conditions were retrieved from the literature to evaluate the model accuracy and active pathways in all the conditions (aerobic and anaerobic conditions). We tested alcohol derivatives, amides, amines, amino acids and derivatives, aromatic compounds, carbohydrates, and nucleotide derivatives as C substrates. In the presence of oxygen, ammonium was set as the sole N nutrient for all C substrates. Besides the C sources validation, we tested 11 organic (amino acids, amino compounds, and nucleotides) and inorganic (ammonium, nitrate, and nitrite) N compounds. During N condition simulations, acetate was used as the main C source. For experimental conditions with no specific uptake rates or experiments with only qualitative results (growth or non-growth), the validation process was reduced to true positive, true negative, false positive, and false negative calls. Furthermore, the model was validated utilizing GN2 Biolog plate results from Allen 2002 [16] to determine the global accuracy of the phenotype predictions. *Thauera sp*. MZ1T could grow in 46 C sources from the GN2 Biolog plates according to the results reported by Allen 2002 under aerobic conditions. Six out of the 46 C sources from these experimental results were not integrated to the M-model due to the lack of evidence about the metabolic pathways in *Thauera sp*. MZ1T to assimilate these compounds (α-ketovaleric acid, bromo succinic acid, liaconic acid, mono-methyl succinate, Tween 40, and Tween 80). For C substrate assessment, ammonium and phosphate uptake rates were not fixed to specific values. Statistical parameters were estimated based on the comparison between the M-model simulations and the experimental values. The model accuracy from the GN2 Biolog plates results was compared with the *in-silico* predictions of other *Thauera sp*. MZ1T models to determine the quality of model simulations. Statistical parameters were calculated to determine model precision, accuracy, positive and negative predictions. Later, the experimental growth values were classified in three categories based on the OD600 measurements of the GN2 Biolog plate results from Allen in 2002: high, medium, and low. The growth rates of the C sources with true positive predictions were distributed in these three categories. The growth values predicted were z-score normalized and sorted following the same three categories from the experimental measurements (high, medium, and low). Based on the model estimations and the experimental results, we categorized the model classifications in three possible outcomes: correct estimation (coincidence between the model prediction and the experimental classification), underestimation (the model estimates a lower category), and overestimation (the model predicts a higher category).

We determined the flux distribution of the reactions per subsystem using the true positive predictions of the GN2 Biolog plates results. Only subsystems with active flux reactions at least in one of the GN2 Biolog plate simulations were included in the analysis. The total flux activity per subsystem was calculated for all the C substrates, and z-score normalized across all the growth conditions to emphasize the flux activity changes. An *in silico* gene essentiality analysis was performed to determine which genes are required for the growth of *Thauera sp*. MZ1T for the GN2 Biolog experimental conditions. We employed the single gene deletion algorithm from the COBRA Toolbox to carry out the gene essentiality analysis. Every gene of the M-model was deleted for every condition and the growth rate value was estimated through FBA after the gene deletion (KO growth rate). All growth rate KOs were compared against the WT growth rate to calculate the growth rate ratio. The genes of the M-model were classified depending on the impact of the knockout on the growth rate value into three groups: non-essential genes, genes with no impact on the growth rate on any of the conditions (growth rate ratio = 1); growth-reducing genes including the model genes with a negative effect in the growth rate (lower than WT growth rate) on any of the growth conditions (1e-6 < growth rate ratio < 1); and lethal genes comprising the genes that decrease the KO growth rate lower than 1e-6 (considering the tolerance of the Gurobi Optimizer Solver) in all of the experimental conditions.

We evaluated the capacity of the M-model to predict the consumption of aromatic compounds under aerobic and anaerobic conditions. The M-model was constrained to consume a single aromatic substrate, ammonium as the sole N source, and all the essential minerals required by *Thauera sp*. MZ1T under aerobic conditions. In the absence of oxygen, ammonium was set as the N source and nitrate replaced oxygen as the main electron acceptor. Additionally, we determined the variation of the metabolic fluxes across the different subsystems in the M-model to identify which subsystems are down and up-regulated using aromatic compounds as C sources. A sampling analysis was executed using the true positive predictions of the aromatic compounds under different oxygen concentrations. The sampling algorithm from the COBRA Toolbox was employed to perform the sampling analysis. The ACHR sampling algorithm was selected, and a total of 5,000 sample points were generated to capture flux variations within the model. The sampling evaluation was performed only for optimal growth values, calculating the internal metabolic fluxes for the maximum biomass production. The sampling results were averaged across samples and compared against the acetate sampling flux distributions through the Mann Whitney U test for aerobic and anaerobic conditions. This non-parametric test allows for robust statistical comparisons without assuming normal distribution of flux data, which enhances interpretability of the metabolic behavior in aerobic and anaerobic conditions. We estimated the up-regulated and down-regulated subsystems per aromatic C source using the p-values of the Mann Whitney U test with acetate as the C substrate reference. The p-values of the Mann Whitney U test were transformed using the logarithmic transformation to highlight the most up-regulated and down-regulated subsystems. Subsystems with less significant p-values were filtered out to maintain only the metabolic pathways with substantial changes compared to the acetate flux distribution.

### 4.4 PHB and EPS *in silico* optimization

We assessed the accuracy of the M-model to predict the PHB production using the experiments of Colpa in 2020 under aerobic N-limiting conditions with acetate as the sole C source [12]. Ammonium was provided as N source with the essential minerals required for *Thauera sp*. MZ1T growth. Different acetate:ammonium ratios were employed to determine the optimal C:N proportion for PHB production. Colpa in 2020 tested 10 g/L of acetate and 0.6 g/L of ammonium in a 10:1 C:N proportion (mol/mol) with an average PHB production of 50% content in cell dry mass. We performed a sensitivity analysis from 1:1 to 20:1 C:N ratios using acetate and ammonium and evaluating a total of 20 scenarios by increasing the C proportion one unit per simulation. PHB production was estimated using the internal flux of the reaction PHB synthase from the M-model. The model was specifically constrained to ensure the production of 50% PHB content and 50% cell dry mass.

Later, we measured the impact of different C substrates and C:N proportions (mol/mol) on the production of PHB and six variants of EPS under different oxygen concentrations (presence or absence). The PHB contribution can achieve values from 15% up to 75% of the total cell dry weight depending on the environmental conditions by *Thauera* and other relevant PHB bacteria producers [57,59,60]. We averaged all the PHB production scenarios to determine the PHB contribution to the final biomass dry weight. We calculated the *in silico* yield of PHB production (initially estimated as production rates in mmol/gDWh) considering an average contribution of 50% in the dry weight (i.e. 50% of biomass production). The yield per C molecule was determined considering the number of carbon molecules present in the chemical formula of each C substrate. The C:N ratios (mol/mol) were estimated considering the C contribution from the C substrate and the N contribution from ammonium and the C substrate when N was part of the C source chemical composition (such as amides, amines, amino acids, and nucleotides). The N contribution was estimated considering ammonium for the aerobic conditions and nitrate for the anaerobic simulations. The estimations were calculated using only the positive predicted C sources from the GN2 Biolog plate experiments in presence and absence of oxygen [16]. The same strategy was utilized to estimate the production per C molecule of six variants of the EPS extracted from the diverse EPS factions characterized by Allen in 2002 and 2016, with a 30% in the dry weight participation in the cell biomass [16,17]. The coefficients for the EPS precursors in each reaction were estimated using the precursor fractions estimated by Allen in 2002 and 2016. The abundance values of each EPS precursor were transformed to determine their contribution to the final EPS structure. The average EPS dry weight participation was estimated considering that bacteria can excrete values from 15% up to 45% of the total cell dry weight depending on the available nutrients and growth conditions. The yield per C molecule for PHB and EPS was compared between the two oxygen scenarios, aerobic against anaerobic production. We performed a Principal Component Analysis (PCA) using the yield of the polymers to identify the effect of the C source and C:N ratio under aerobic and anaerobic conditions. The results of the PCA distributions were clustered based on the C sources, C:N ratio values, and the oxygen concentration (presence or absence).

Ultimately, we evaluated the essential reactions involved in the production and efficiency of PHB and EPS in *Thauera sp*. MZ1T. A reaction essentially analysis was executed to determine the relevance of each reaction and the associated TMZ genes on the growth rate using the experimental conditions of GN2 Biolog plates in two oxygen scenarios (presence and absence). The pseudo reactions created only for modeling purposes were not included in the single deletion analysis such as exchange reactions. Reactions evaluated in the deletion analysis were classified based on their GPR associations, generating two categories: 1) reactions with TMZ genes and 2) orphan reactions. Every reaction of the M-model was deleted for every condition and the growth rate was estimated through FBA after the reaction deletion. The reactions were grouped depending on the impact of the reaction removed from the M-model on the growth rate value: non-essential reactions (growth rate ratio = 1); growth-reducing reactions (1e-6 < growth rate ratio < 1); and lethal reactions (growth rate ratio < = 1e-6). Additionally, we identified the subsystems which the lethal reaction belongs to under aerobic and anaerobic conditions. The biological role of the enzymes related to the lethal reactions were determined based on the annotation of *Thauera sp*. MZ1T.

## Supporting information

S1 Material List of 391 manually curated reactions utilized to calibrate the sensitivity analysis in the initial reconstruction process. (XLSX)

S2 Material Venn Diagram comparing the common reactions in the three template models and in iThauera861. (SVG)

S3 Material Detail lists of the common reactions observed in the three template models and in iThauera861. (XLSX)

S4 Material Carbon and nitrogen substrates employed for phenotyping using GN2 Biolog plates including the experimental and simulation outcomes. (XLSX)

S5 Material Subsystem flux distributions for different carbon sources under aerobic conditions. (DOCX)

S6 Material Effect of the C:N ratio and different carbon sources on production of PHB and EPS under anaerobic conditions. (DOCX)

S7 Material Gene essentiality analysis for the production of PHB and EPSs under aerobic and anaerobic conditions using different carbon sources. (DOCX)

S8 Material Diagram with the subsystem’s contributions from each template model and the specific *Thauera* sp. MZ1T metabolic pathways.
(DOCX)
