# Supplementary material for: A genome-scale metabolic model for the denitrifying bacterium Thauera sp. MZ1T accurately predicts degradation of pollutants and production of polymers
Source: PLoS Comput Biol. 2025 Jan 7;21(1):e1012736. doi: 10.1371/journal.pcbi.1012736 (PMC11741664; doi:10.1371/journal.pcbi.1012736)
Supplement: S5 Material — (DOCX) [file pcbi.1012736.s005.docx]

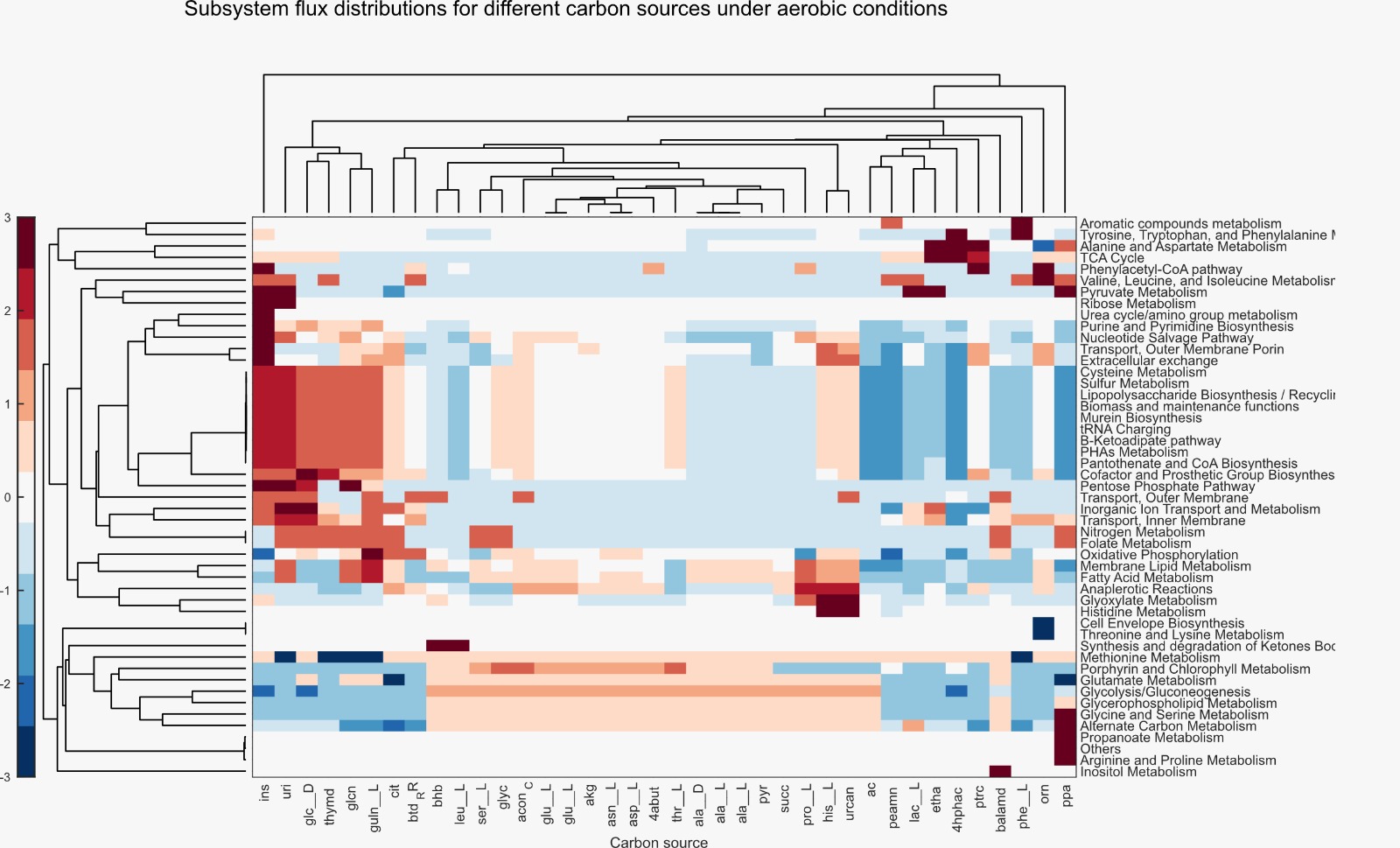


**S2 Fig. Subsystem flux distributions for different carbon sources under aerobic conditions.** Total flux accumulation per subsystem across the 36 carbon sources determined by FBA. Flux distributions were z-score normalized (-3 to 3) and displayed in a clustergram. 49 out of the 57 subsystems were active in at least one experimental condition. Some amino acids like asparagine (asn__L), aspartate (asp__L), and glutamine (gln__L) presented similar flux distributions across most of the subsystems. Other amino acids like histidine (his__L) and phenylalanine (phe__L) had completely different trends from the rest of the amino acids. Abbreviations: ins, inosine; uri, uridine; glc__D, D-glucose, thymd, thymidine; glcn, D-gluconic acid; guln__L, gulonic acid; cit, citric acid; btd_RR, 2,3- butanediol; bhb, β-hydroxybutyric acid; leu__L, L-leucine; ser__L, L-serine; glyc, glycerol; acon_C, cis-aconitic acid; glu__L, L-glutamic acid; akg, α-ketoglutaric acid; asn__L, L-asparagine; asp__L, L-aspartic acid; 4abut, γ-amino butyric acid; thr__L, L-threonine; ala__D, D-alanine; ala__L, L-alanine; pyr, pyruvic acid; succ, succinic acid; gln__L; L-glutamine; pro__L, L-proline; his__L, L-histidine; urcan, urocanic acid; ptrc, putrescine; 4hphac, β-hydroxyphenylacetic acid; etha, 2-amino ethanol; lac__L, L-lactic acid; peamn, phenyl ethylamine; ac, acetate; balamd, alaninamide; phe__L, L-phenylalanine; orn, L-ornithine; ppa, propionic acid.
