# Supplementary material for: A genome-scale metabolic model for the denitrifying bacterium Thauera sp. MZ1T accurately predicts degradation of pollutants and production of polymers
Source: PLoS Comput Biol. 2025 Jan 7;21(1):e1012736. doi: 10.1371/journal.pcbi.1012736 (PMC11741664; doi:10.1371/journal.pcbi.1012736)
Supplement: S6 Material — (DOCX) [file pcbi.1012736.s006.docx]

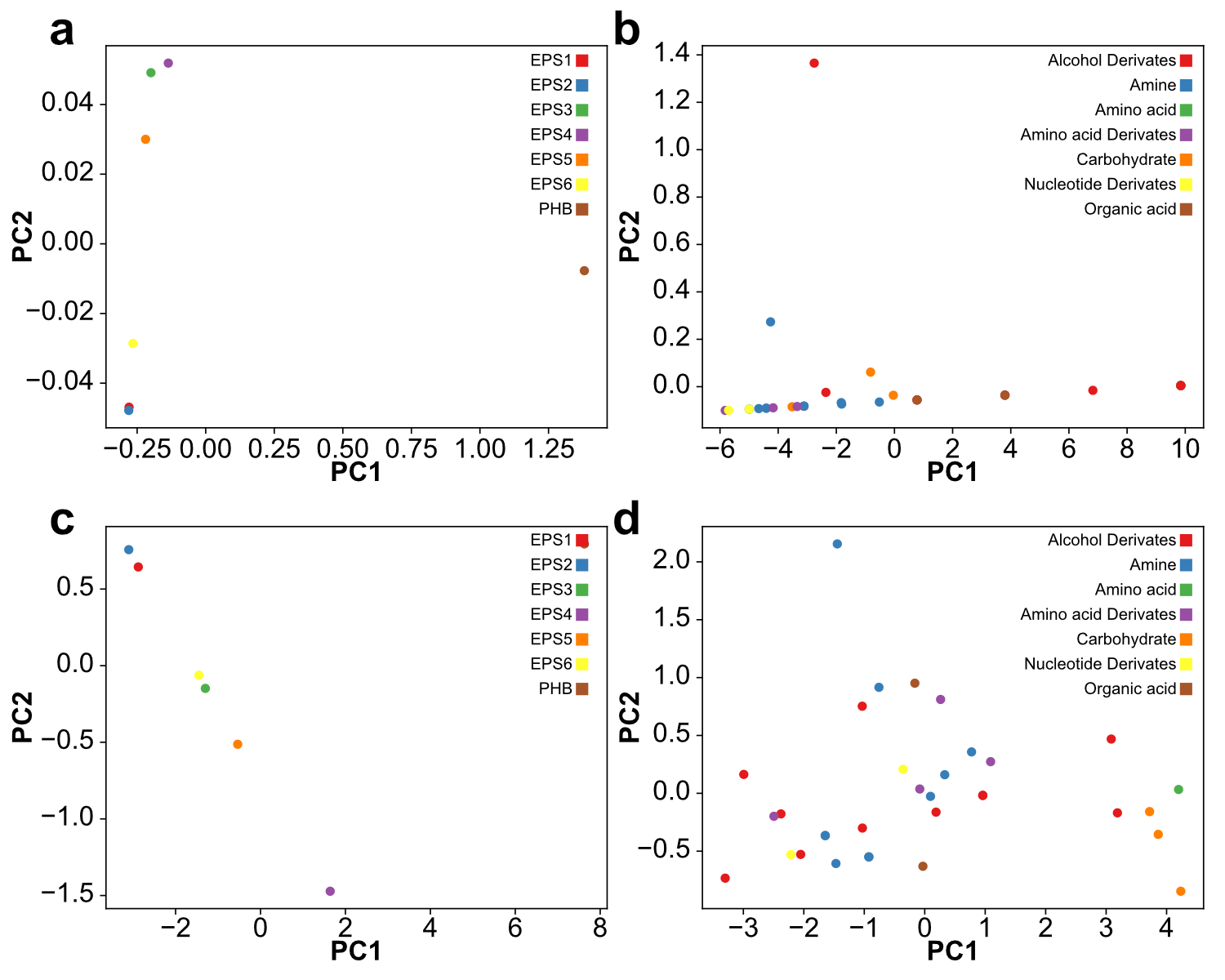


**S2 Fig. Effect of the C:N ratio and different carbon sources on production of PHB and EPS under anaerobic conditions.** **A** PCA plot displaying the two first component scores of the yield per C molecule of PHB and EPS considering the PCA coefficients of PHB and EPS under aerobic conditions with 34 carbon sources. The PCA represents 99% of the variability of the data. Two main clusters can be identified, the cluster of the EPS (left side) and the cluster of the PHB (right side). **B** First two component scores of the yield per C molecule of PHB and EPS PCA under anaerobic conditions, considering the PCA coefficients of C sources. The compounds were grouped by their common functional groups. No clear clustering was observed based on the C sources. **C** PCA plot displaying the two first component scores of the C:N ratios considering the PCA coefficients of PHB and EPS under anaerobic conditions. The PCA represents 99% of the variability of the data. Two main clusters can be identified, the cluster of the EPS (left side) and the cluster of the PHB (right side). **D** First two component scores of the C:N ratio PCA under anaerobic conditions, considering the PCA coefficients of C sources. The compounds were grouped by their common functional groups. No clear clustering was observed based on the C sources based on the C:N ratio component scores.
