# Supplementary material for: A genome-scale metabolic model for the denitrifying bacterium Thauera sp. MZ1T accurately predicts degradation of pollutants and production of polymers
Source: PLoS Comput Biol. 2025 Jan 7;21(1):e1012736. doi: 10.1371/journal.pcbi.1012736 (PMC11741664; doi:10.1371/journal.pcbi.1012736)
Supplement: S7 Material — (DOCX) [file pcbi.1012736.s007.docx]

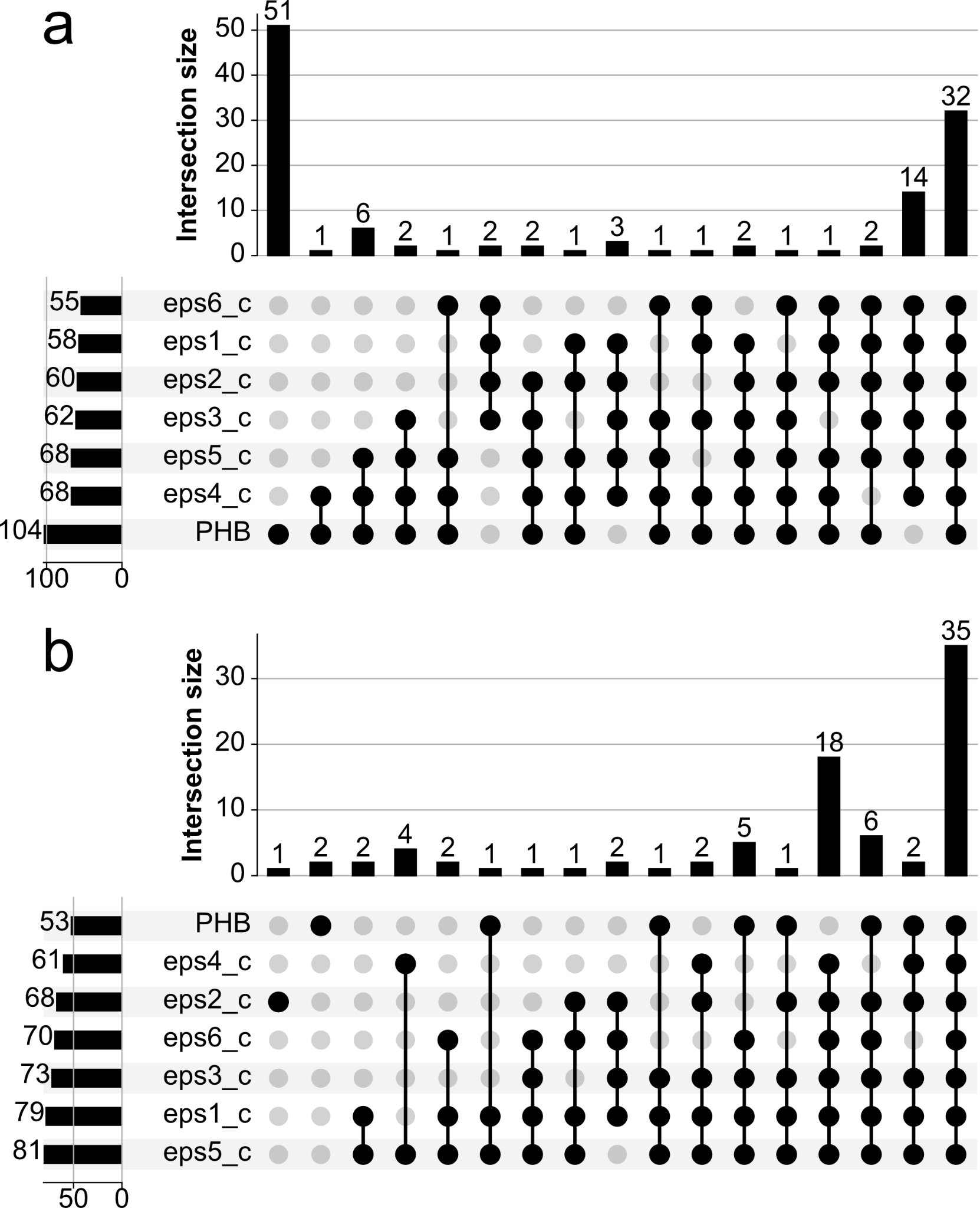


**S3 Fig. Gene essentiality analysis for the production of PHB and EPSs under aerobic and anaerobic conditions using different carbon sources.** **A** Upset plot with 20 distinct groups of lethal genes for PHB and six EPSs under aerobic conditions. **B** Upset plot with 19 diverse groups of lethal genes for PHB and six EPSs under anaerobic conditions.
