# Supplementary material for: A genome-scale metabolic model for the denitrifying bacterium Thauera sp. MZ1T accurately predicts degradation of pollutants and production of polymers
Source: PLoS Comput Biol. 2025 Jan 7;21(1):e1012736. doi: 10.1371/journal.pcbi.1012736 (PMC11741664; doi:10.1371/journal.pcbi.1012736)
Supplement: S8 Material — MZ1T metabolic pathways. (DOCX) [file pcbi.1012736.s008.docx]

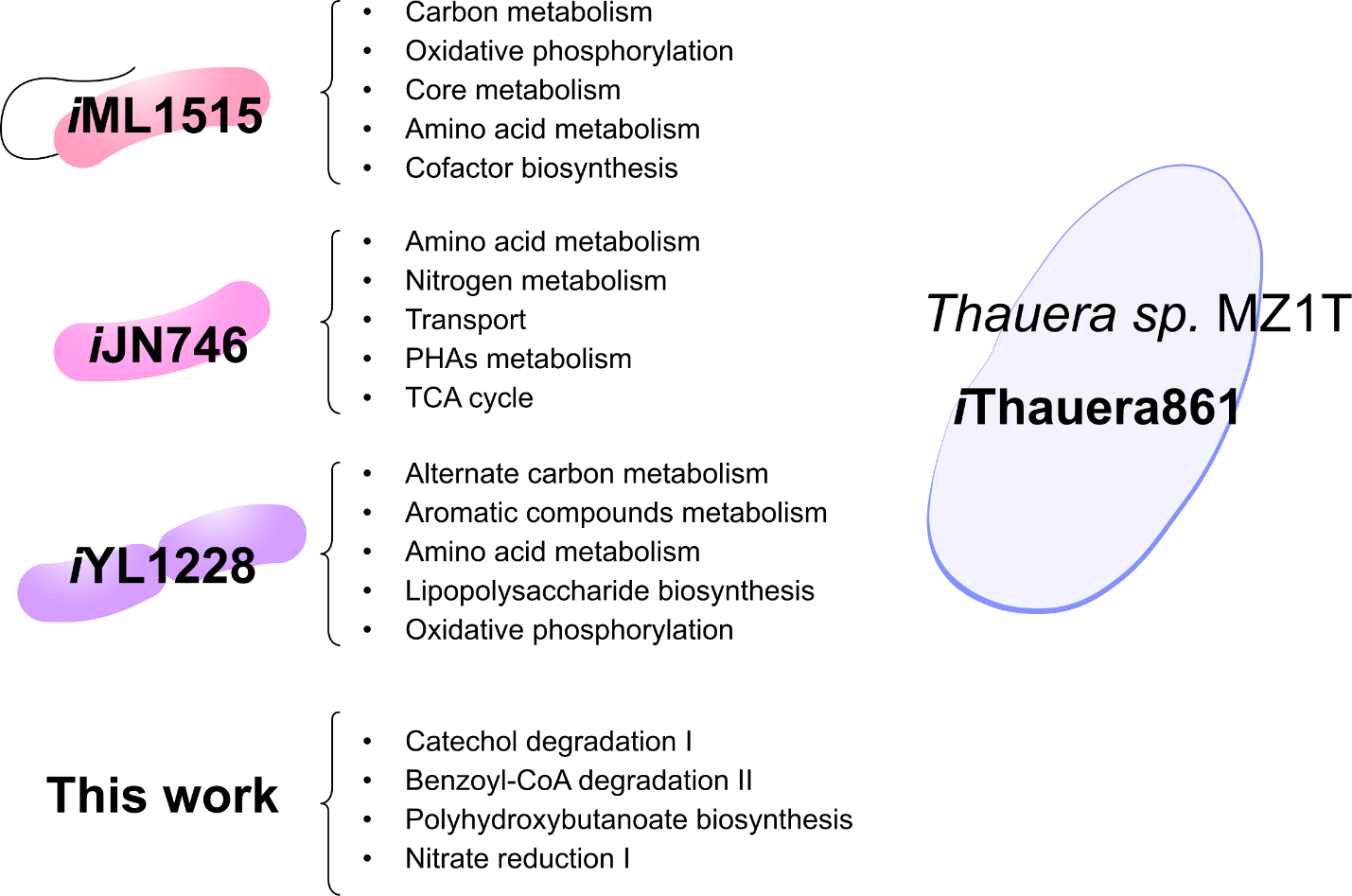


S8 Material. Diagram with the subsystem’s contributions from each template model and the specific *Thauera* sp. MZ1T metabolic pathways.
